# Insights into mechanisms of MALT1 allostery from NMR and AlphaFold dynamic analyses

**DOI:** 10.1038/s42003-024-06558-y

**Published:** 2024-07-16

**Authors:** Johan Wallerstein, Xiao Han, Maria Levkovets, Dmitry Lesovoy, Daniel Malmodin, Claudio Mirabello, Björn Wallner, Renhua Sun, Tatyana Sandalova, Peter Agback, Göran Karlsson, Adnane Achour, Tatiana Agback, Vladislav Orekhov

**Affiliations:** 1 Department of Chemistry and Molecular Biology, University of Gothenburg, Box 465, SE-40530 Gothenburg, Sweden; 2 Science for Life Laboratory, Department of Medicine, Solna, Karolinska Institute, SE-17165 Solna, Sweden; 3 Division of Infectious Diseases, Karolinska University Hospital, SE‑171 76 Stockholm, Sweden; 4 Swedish NMR Centre, University of Gothenburg, Box 465, SE-40530 Gothenburg, Sweden; 5 Shemyakin-Ovchinnikov Institute of Bioorganic Chemistry RAS, 117997 Moscow, Russia; 6 Dept of Physics, Chemistry and Biology, Linköping University, 581 83 Linköping, Sweden; 7 National Bioinformatics Infrastructure Sweden, Science for Life Laboratory, Solna, Sweden; 8 Department of Molecular Sciences, Swedish University of Agricultural Sciences, PO Box 7015, SE-750 07 Uppsala, Sweden

**Keywords:** Solution-state NMR, B-cell lymphoma

## Abstract

Mucosa-associated lymphoid tissue lymphoma-translocation protein 1 (MALT1) is an attractive target for the development of modulatory compounds in the treatment of lymphoma and other cancers. While the three-dimensional structure of MALT1 has been previously determined through X-ray analysis, its dynamic behaviour in solution has remained unexplored. We present here dynamic analyses of the apo MALT1 form along with the E549A mutation. This investigation used NMR ^15^N relaxation and NOE measurements between side-chain methyl groups. Our findings confirm that MALT1 exists as a monomer in solution, and demonstrate that the domains display semi-independent movements in relation to each other. Our dynamic study, covering multiple time scales, along with the assessment of conformational populations by Molecular Dynamic simulations, Alpha Fold modelling and PCA analysis, put the side chain of residue W580 in an inward position, shedding light at potential mechanisms underlying the allosteric regulation of this enzyme.

## Abstract (web summary)

NMR relaxation and AlphaFold structural ensemble modelling of MALT1 reveals motions between its PCASP and Ig3 domains. This sheds light into the mechanisms of the protein’s allosteric regulation.

## Introduction

The human mucosa-associated lymphoid tissue lymphoma-translocation protein 1 (MALT1) is a unique human paracaspase recognized for its distinct cleaving capacity and substrate specificity^1^. Crucial for the survival, proliferation and functions of B and T cells in adaptive immune responses, MALT1 activates the NF-κB signalling pathway following antigen stimulation^2–5^. Dysregulated MALT1 activity is implicated in various lymphoid malignancies, leukaemia^6–8^ and several other cancers including glioblastoma, melanoma, and breast cancer^9–13^, as well as a large array of autoimmune diseases^14–18^. As a key regulator of adaptive immune responses, MALT1 represents a clear target for developing modulatory/inhibitory compounds to treat lymphoma, cancers, and autoimmune diseases.

**Fig. 1:**
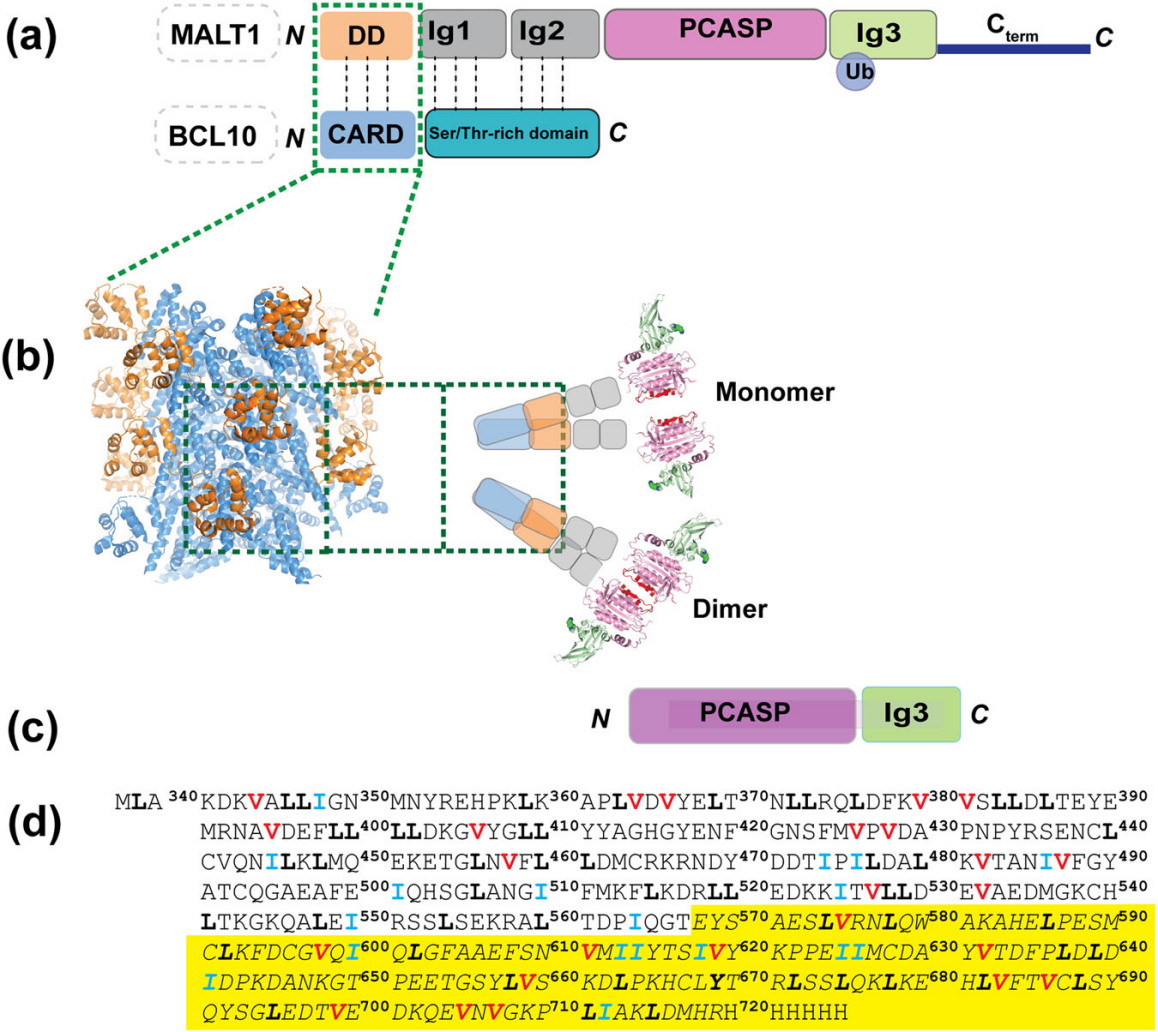
The MALT1-BCL-10 complex. **a** Protein domain structure: MALT1 forms a complex with the protein BCL10 through interactions between the DD and CARD domains. Sites for mono- and poly-ubiquitination are indicated. **b** The 4.5 Å cryo-EM model of the MALT1-BCL10 complex was created by PyMol (http://www.pymol.org/pymol) from the filament structure (PDB code 6GK2)^25^ reveals a filament core formed by BCL10 (blue) into which the MALT1 DD domain (orange) is inserted. Notably, none of the other domains of MALT1 are resolved in the cryo-EM study due to their high flexibility. It has been proposed that the putative arrangement of the MALT1 complex is in equilibrium between monomer and dimer forms. **c** MALT1 construct used within this study. **d** Sequence of the MALT1(PCASP-Ig3)_339–719_ construct, with the methyl-containing residues I, L and V labelled in blue, black and red, respectively, and the sequence covering the Ig3 domain in yellow.

By forming filament CBM complexes alongside BCL10 and CARD11^19–21^, MALT1 serves both as a scaffolding template and a protease, recruiting signalling factors and cleaving substrate proteins to regulate *e.g*. T cell activation^22–24^. MALT1 exhibits a complex multi-domain structure comprising an N-terminal death domain (DD), followed by the two immunoglobulin domains Ig1 and Ig2, the paracaspase domain (PCASP), and a C-terminal Ig3 domain (Fig. 1a). Recent cryo-EM analyses^25^ unveiled that BCL10 forms filaments adorned by MALT1, with specific emphasis on the incorporation of the DD domain. However, the structural flexibility of the remaining MALT1 protein precluded its visualisation, leaving the exact conformational ensembles of these parts of the formed complexes unknown (Fig. 1b)^25^.

MALT1, initially identified as a distant relative of caspases during efforts to trace their evolutionary origin^26^, has been postulated to exist as an inactive monomer that becomes activated through oligomerization/dimerization in the CBM complex (Fig. 1b). It has been demonstrated that MALT1 constructs, initially obtained as monomers through size exclusion chromatography under physiological salt concentrations, show a tendency to spontaneously form dimers in solution^27^. This finding aligns well with the concept that dimerization is crucial for the formation of an active MALT1 complex^27,28^. In vitro studies provided further support for the significance of dimerization in MALT1 activation. Notably, elevated MALT1 activity was observed in the presence of the kosmotropic ammonium citrate buffer^24,29,30^, known for activating initiator caspases by promoting their dimerization^31,32^, which pinpoints the crucial role of dimerization in MALT1 activation. However, this enhanced activity was completely abolished in the E549A mutant, affecting both MALT1 dimerization and activity^33^.

To elucidate how the formation of the CBM complex activates MALT1, pioneering biochemical and crystallographic studies have been previously conducted in vitro^27,28^. Truncated ligand-free constructs of MALT1, encompassing the PCASP and the adjacent Ig3 domain (MALT1(PCASP-Ig3)_339–719_ (Fig. 1c)), were purified and separated into monomeric and dimeric forms using size-exclusion chromatography for subsequent structural analysis. The three-dimensional structure of the dimeric form was determined to 1.8 Å resolution (PDB code 3V55), revealing an inactive self-inhibited configuration^27^. Notably, no MALT1 monomers were observed to crystallise under these conditions^27,28^.

The PCASP domain of MALT1 harbours the histidine/cysteine catalytic dyad H415/C464 within its active centre (Fig. 1a), that triggers cleavage activity in T lymphocytes upon antigen receptor engagement^23,24^. The catalytic activity of PCASP has been previously established, with a particular specificity towards peptide substrates featuring an arginine residue at position 1 (PDB code 3V4O)^29,34^. The crystal structure of an active conformation has also been previously determined, as an irreversibly modified dimer of MALT1(PCASP-Ig3)_339–719_ in complex with a substrate-mimicking inhibitor^23,27,28^. It was concluded from these pioneering studies that the transition from the inactive to active conformation of MALT1, which was induced by the inhibitor, involves profound structural changes propagating throughout the PCASP domain, particularly affecting the active site and dimerization interface, along with a long-range of structural alterations. Notably, allosteric conformational changes were introduced in the Ig3 domain during PCASP activation and dimerization, a phenomenon that seemed to be accentuated by binding of the inhibitor to the active site.

Mono-ubiquitination of MALT1 highlights an alternative allosteric activation pathway connecting the PCASP and Ig3 domains^30,33,35^. Ubiquitin (Ubq) binds to Ig3 via a negatively charged surface patch, including residues E696 and D697, opposite the Ig3-protease domain interaction surface^35^. Mutation of Y657 in Ig3 induces coordinated conformational changes in the loop connecting K644 to Y657, and the active site. This mutational result led to the proposal that Ubq’s covalent attachment to K644 may structurally alter the loop extending from K644 to Y657, potentially affecting hydrophobic inter-domains interactions, such as those between Y657 on Ig3, and both L506 and Y367 in PCASP. These interactions were notably altered upon binding of the substrate analogue z-VRPR-fmk to the active site of MALT1^28^. Emphasising the role of Y657 as a key mediator in signalling between Ig3 and protease domains, this previous study suggested that Ubq attachment to K644 induces allosteric conformational changes in the Ig3-protease interface, ultimately enhancing MALT1 activation.

These previous studies explored also the complex network of allosteric communication within and between different protein domains in MALT1(PCASP-Ig3)_339–719_. Allostery which is a phenomenon that couples a ligand binding at one site of a protein with a conformational or dynamic change at a distant site, is crucial for biological signalling. A thorough understanding and ultimately potential targeting of such regulatory allosteric pathways could represent a key step for the adequate design of new high specificity drugs and disease treatments. Progress has been made in identifying a specific allosteric target pocket localized between the PCASP and Ig3 domains. This advancement has facilitated the development of compounds that inhibit MALT1 function in a non-competitive, allosteric manner^1,36,37^. However, relying solely on a static structural view^38^ is insufficient, and several challenges persist in identifying and characterising other functional allosteric sites in MALT1. Previous studies on the nature of allostery^39,40^ suggest that long-range communication is often not limited to changes in the mean protein conformation, but involve also a redistribution of structural ensembles, and changes in amplitudes and time scales of dynamic fluctuations about the mean conformation^41–44^.

NMR spectroscopy provides a unique possibility to investigate how protein motions contribute to the transmission of allosteric signals^41,45–51^. It offers important insights into structural details, allows the characterisation of molecular motions across various time scales^50,52,53^, provides access to sparsely populated conformational states^50,54^, and to residue-specific information about conformational entropy^39,55,56^. From this perspective, allosteric transitions are embedded within the composition of structural ensembles, whose relative populations can be modulated under different conditions^39,57^. Thus, the ensemble allosteric model postulates that the potential for various allosteric mechanisms is inherently pre-encoded in protein structure ensembles^39,58^.

Long molecular dynamics (MD) simulations in explicit solvent and modelling utilising such advanced methods such as AlphaFold (AF)^59^ are methods that can be used to create conformational ensembles at atomic resolution. Additionally, even though AF was initially designed to predict three-dimensional protein structures with a high quality comparable to those obtained using experimental methods^59^, recent advancements demonstrated also its capability to generate multiple conformations and predict dynamic regions at the individual residue level^60–63^.

Despite numerous publications on MALT1 over the past decade, to our knowledge, there have been no reports of NMR dynamic studies on the paracaspase MALT1(PCASP-Ig3)_339–719_ or similar constructs. In this study, we investigated the dynamics of MALT1(PCASP-Ig3)_339–719_ and the E549A mutated variant in solution. We focused our study on the truncated MALT1(PCASP-Ig3)_339–719_ because it is a minimal unit exhibiting both proteolytic activity and allosteric regulation mediated by the dynamics at the interface of PCASP and Ig3 domains. This allosteric regulation may be important for balancing the dual protein function as both a protease and a scaffold for recruiting other proteins. Therefore, we hypothesized that the switch between these two functions of MALT1 in vivo could be explicitly regulated by the dynamics at the interface between PCASP and Ig3 domains.

We have previously presented the almost complete assignment of the ^15^N/^13^C/^1^H backbone^64^ and of the IVL-Methyl side chains^65^ for the apo form of human MALT1(PCASP-Ig3)_339–719_ using high-resolution NMR. By using ^15^N-backbone *R*_1_- and *R*_2_-relaxation, and ^15^N cross-correlated transverse relaxation (CCR) rates, we here delved into crucial information concerning overall rotational diffusion, internal dynamics, and inter-domain motion. Additionally, we employed the recently developed AF and an array of other structure modelling methods to identify and characterise conformational ensembles for MALT1(PCASP-Ig3)_339–719_, which we thereafter used to assess the allosteric pathways using the principal component analysis (PCA).

## Results

### MALT1(PCASP-Ig3)_339–719_ forms a monomer in solution

Several three-dimensional structures have been reported for different separate regions of the multidomain MALT1^27,28,66^. The low-resolution cryo-EM model of the BCL10-MALT1 complex^25^ (Fig. 1b**)** unveiled the organisation of MALT1 N-terminal DD domains within the CBM filament. However, this cryo-EM structure did not provide any information about the configuration of the other regulatory and PCASP domains, likely due to their inherent conformational dynamics. All the previously determined crystal structures of MALT1(PCASP-Ig3)_339–719_ (Fig. 1c) in the apo-form or in complex with ligands have the same dimerization interface of about 1000Å^2^ (Fig. 1b). Up to now, there is to our knowledge no available three-dimensional structure of the monomeric form of MALT1(PCASP-Ig3)_339–719_ in solution.

NMR provides an alternative way, compared to gel filtration analyses, to discriminate monomeric and multimeric protein forms in solution, through the direct estimation of the rotational correlation time *τ*_C_. The experimental *τ*_C_ can be compared with the values predicted using an empiric equation that takes into account the temperature and molecular weight^67^. Such estimates gave *τ*_C_ values of 26.6 ns and 52.9 ns for the monomeric and dimeric forms of MALT1(PCASP-Ig3)_339–719_, respectively. The former value agrees well with our experimental results (Table 1), thus confirming the predominantly monomeric form of MALT1(PCASP-Ig3)_339–719_ in solution. Another way to address this topic is to compare the NMR-derived *τ*_C_ values for the wild-type and the E549A-mutated forms of MALT1(PCASP-Ig3)_339–719_. The mutation which is located within the dimerization interface of MALT1(PCASP-Ig3)_339–719_ prevents dimerization and stabilises the molecule in its monomeric state in solution^33^.

**Table 1:** Hydrodynamic characteristics of the PCASP and Ig3 domains in MALT1(PCASP-Ig3)_339–719_

Data set	Domain	# Res	*τ*_C_^a^	D_⊥_^b^	D_||_^b^	Anisotropy^c^	α^d^	β^d^
900 MHz R_2_/R_1_	PCASP	55	27.4 ± 1.5	0.52 ± 0.02	0.78 ± 0.04	1.48 ± 0.05	18	43
	Ig3	55	25.5 ± 1.5	0.62 ± 0.02	0.72 ± 0.04	1.17 ± 0.05	47	131
	full	116	26.5 ± 1.2	0.56 ± 0.02	0.77 ± 0.03	1.37 ± 0.04	11	59
800 MHz R_2_/R_1_	PCASP	57	28.3 ± 1.9	0.52 ± 0.02	0.74 ± 0.04	1.43 ± 0.05	15	41
	Ig3	52	26.8 ± 2.5	0.57 ± 0.04	0.73 ± 0.05	1.28 ± 0.06	14	62
	full	115	27.1 ± 1.7	0.56 ± 0.03	0.73 ± 0.04	1.32 ± 0.05	7	64
900 MHz CCR	PCASP	44	28.0 ± 2.8	0.54 ± 0.03	0.70 ± 0.06	1.29 ± 0.07	N/A	N/A
	Ig3	52	27.8 ± 2.1	0.56 ± 0.03	0.68 ± 0.05	1.22 ± 0.06	N/A	N/A
	full	105	27.8 ± 1.5	0.55 ± 0.02	0.69 ± 0.04	1.25 ± 0.05	N/A	N/A

^a^ Axially symmetric rotational correlation time (in ns) of the molecule. Error propagated from *D*_||_ and *D*_⊥_ using Eq. (3).

^b^ Principal values (in 10^7 ^s^–1^) of the rotational diffusion tensor.

^c^ Degree of anisotropy (ζ) of the diffusion tensor ζ = *D*_||_ / *D*_⊥_.

^d^ The angles cannot be reliably calculated in the resampling procedure for the 900 MHz CCR data due to poor data quality.

First, we investigated whether the E549A mutation triggers any conformational changes in the PCASP domain, which should be revealed by differences in peak positions in the ^1^H-^15^N TROSY spectra for the apo form of MALT1(PCASP-Ig3)_339–719_ and the E549A variant (Supplementary Fig. S1). Comparison of the spectra revealed that the largest changes in the chemical shifts were observed only in the vicinity of the mutation site, indicating an expected alteration in local environments rather than extensive conformational changes in the mutated protein. Secondly, we compared the cross correlated transverse relaxation rates *η*_xy_ of the protein amide groups in MALT1(PCASP-Ig3)_339–719_ (E549A) and wild-type MALT1(PCASP-Ig3)_339–719_ (Supplementary Fig. S2d). The *η*_xy_ values observed for the two molecules are statistically very similar. Furthermore, since *η*_xy_ is related to the apparent residue-specific rotational correlation time *τ*_C_, as described in Eq. (4), it follows that the values of *τ*_C_ are also similar between MALT1(PCASP-Ig3)_339–719_(E549A) and wild-type MALT1(PCASP-Ig3)_339–719_ (Table 1). Noteworthy is that the overall correlation time of MALT1(PCASP-Ig3)_339–719_ estimated from *η*_xy_ values is in agreement with the ‘empirically’ predicted values for the monomer. This finding supports our conclusion that the overall correlation time of MALT1(PCASP-Ig3)_339–719_ corresponds to a monomer as the main form in solution.

### Contribution of the slow chemical exchange in the transverse relaxation R2 of backbone

The conformational exchange *R*_*ex*_, occurring in the protein at rates ranging from tens to thousands of inverse seconds can contribute considerably to the measured transverse relaxation rates *R*_*2*_ (Eq. 1b). Consequently, *R*_*ex*_ can exert a notable influence on the *R*_*2*_*/R*_*1*_ analysis of molecular rotational diffusion. In our efforts to exclude residues that exhibited a statistically significant *R*_*ex*_ contribution to *R*_*2*_ from the below discussed *R*_*2*_*/R*_*1*_ analysis, we calculated *R*_*ex*_ values using Eq. (6).

**Fig. 2:**
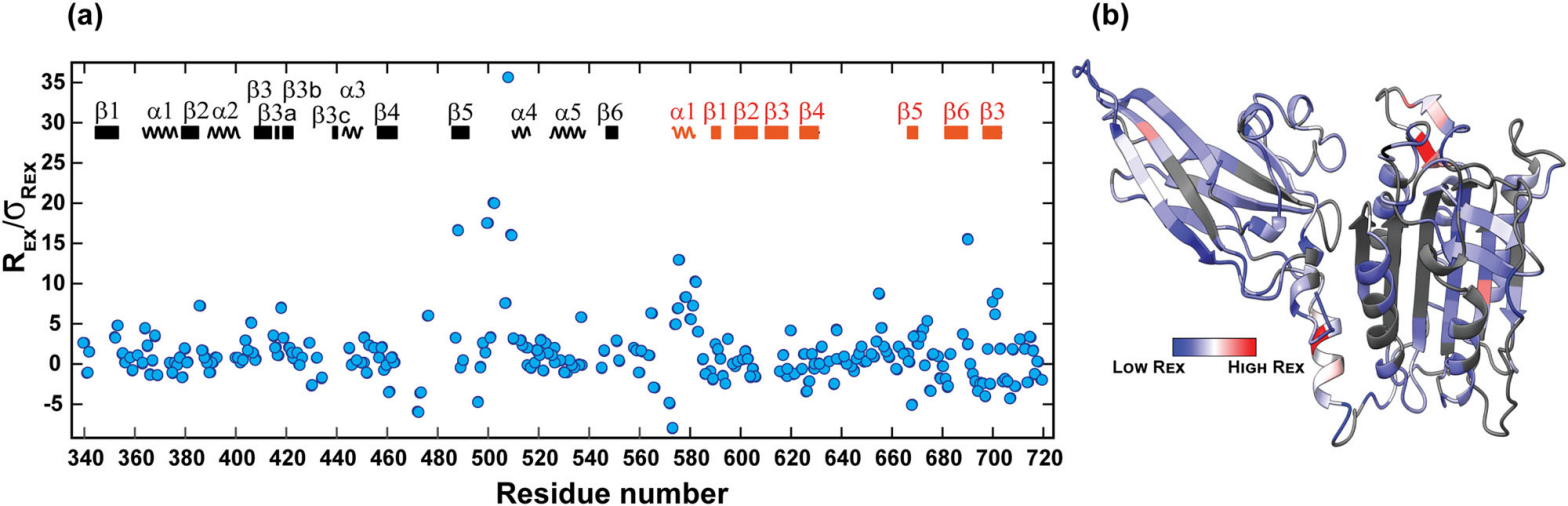
Conformational exchange in milli- micro-second time scale. **a** *R*_ex_/σ_*R*ex_ ratios. The secondary structure elements are indicated and numbered for each domain with PCASP and Ig3 in black and red, respectively. **b** *R*_ex_ values from the 900 MHz data were mapped on the crystal structure of MALT1(PCASP-Ig3)_339–719_.

The most substantial exchange was observed in three specific regions corresponding to the stretches of residues 470–484, 492–510 and 560–590 (Fig. 2a). These stretches are localized within the extensive loop region in the MALT1 dimerization interface, comprising the interdomain linker including the α1-helix, and the region near the active site in the PCASP domain. Importantly, this observed exchange corresponds very well to regions in which residues are either absent or exhibit high B-factors in the crystal structures of MALT1(PCASP-Ig3)_339–719_ (Fig. 2a, b). Another important observation is that the baseline values of *R*_ex_/σ_*R*ex_ for all residues, except those involved in *R*_*ex*_, exhibit remarkable similarity between the PCASP and Ig3 domains. This observation is important for the analysis of individual diffusion tensors presented here below.

### Determination of rotational diffusion parameters from relaxation data

**Fig. 3:**
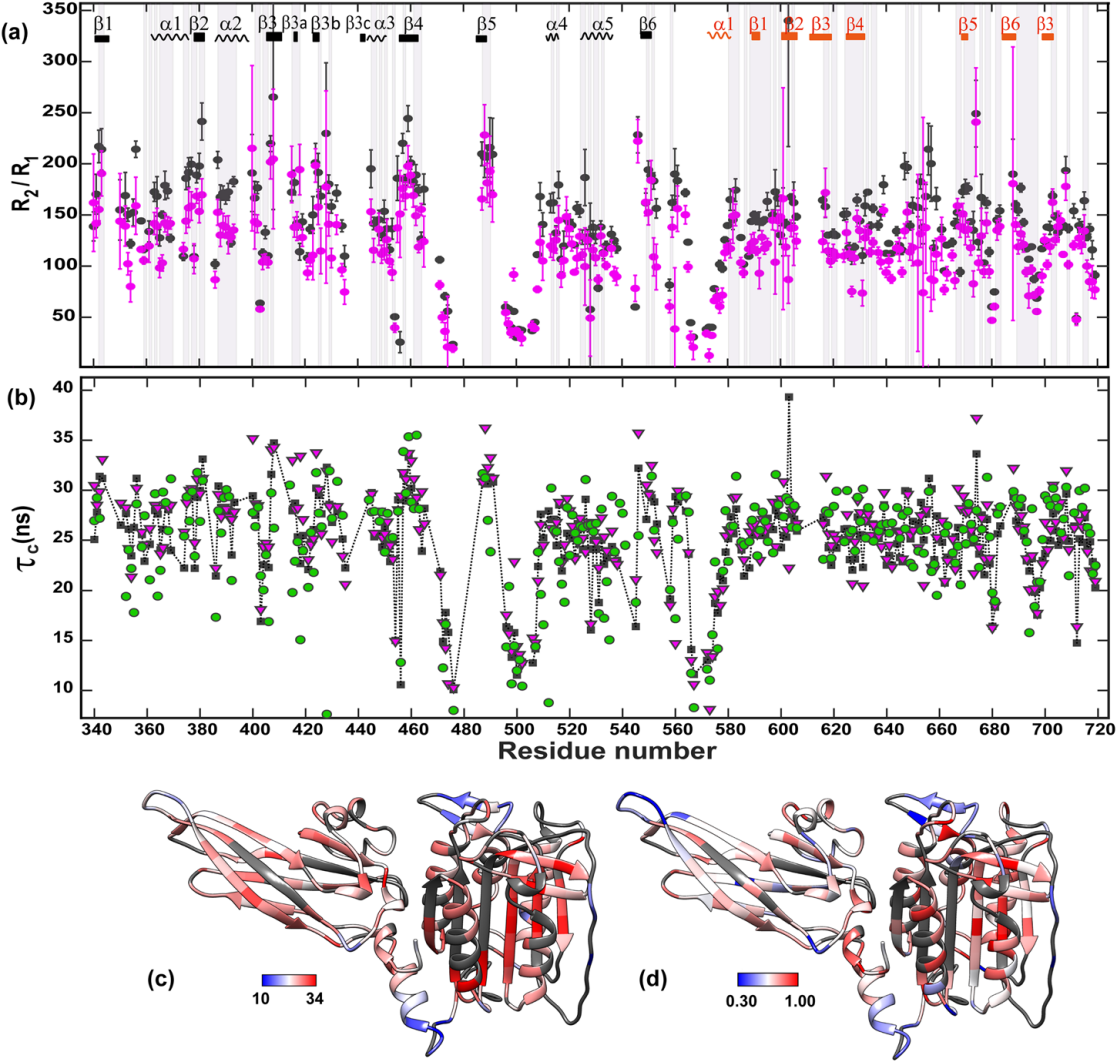
Residue specific dynamics in the nano-picosecond time scale. **a** ^15^N *R*_2_/*R*_1_ ratios for MALT1(PCASP-Ig3)_339–719_. Black and magenta dots represent 900 and 800 MHz data, respectively. Secondary structure elements are indicated and numbered for each domain at the top of the panel, with PCASP in black and Ig3 in red. α1 at residue 574 marks the division between the PCASP and Ig3 domains. The vertical shaded bars indicate the residues used in diffusion tensor calculation (Table S1). Residues highlighted by grey vertical bars in (**a**) are the 116 selected residues that were used for the hydrodynamic calculations for 900 MHz data. Error bars show one SD. **b** Local correlation time per residue *τ*_C,i_ for MALT1(PCASP-Ig3)_339–719_ calculated from the 900 MHz ^15^N *R*_2_/*R*_1_ ratio (black square), 800 MHz ^15^N *R*_2_/*R*_1_ ratio (magenta triangle) and cross-correlated transverse relaxation (green circles). *τ*_C,i_ were calculated from Eqs. 1 and 7. **c** Local effective correlation time ranging from 8 to 37 ns, from 900 MHz ^15^N *R*_2_/*R*_1_ mapped on the crystal structure of MALT1(PCASP-Ig3)_339–719_ (PDB code 3V55). **d** Heteronuclear NOE ranging from 0.3 to 1.0, from the 800 MHz data mapped on the crystal structure of MALT1(PCASP-Ig3)_339–719_. The ^15^N-(^1^H)NOE data are presented in Supplementary Fig. S2c. Residues in dark grey are unassigned or not used in the analysis.

Global tumbling provides important insights into the size and shape of proteins in solution. This aspect is especially pertinent in the case of protein homo and hetero complexes and, as demonstrated in the present study, can even shed light on interdomain motions of multidomain proteins^68^. The process that we used to obtain reliable diffusion tensors is explained in the material and methods section. Figure 3 presents the ratios of the transverse and longitudinal relaxation rates *R*_2_/*R*_1_, which are used for calculations of the diffusion tensors as well as the effective local per-residue rotational correlation times *τ*_C,i_ (Fig. 3b, c) and heteronuclear ^1^H-^15^N NOEs (Supplementary Fig. S2a, b and Supplementary Tables S1–3). The *τ*_C,i_ and/or the ^1^H-^15^N NOE values, depicted in Fig. 3c, d, indicate the regions (*i.e*. the α1 helix and the three loops close to the catalytic site) that display significant mobility in the pico-nano second time range. It should be noted that the corresponding residues were not used in the diffusion tensors calculations.

**Fig. 4:**
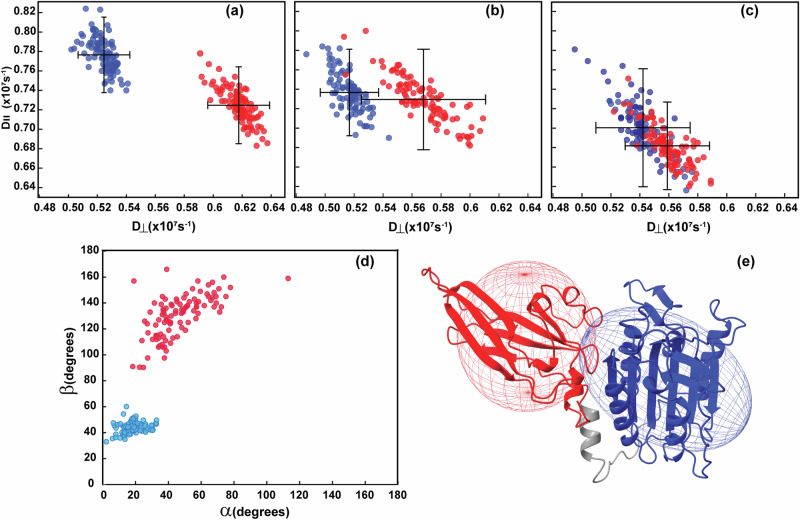
Diffusion tensor components *D*_||_ and *D*_⊥_ from resampled ROTDIF optimisations. Each red and blue dot corresponds to a tensor calculated for one resampling instance for the Ig3 and PCASP domains, respectively, for (**a**) 900 MHz ^15^N *R*_2_/*R*_1_ data, (**b**) 800 MHz ^15^N *R*_2_/*R*_1_ data, (**c**) 900 MHz and cross-correlated transverse relaxation data. Error bars in (**a**–**c**) show one SD and are calculated taking the resampling fraction of *d* = 20% into account. **d** *α* and *β* angles for the diffusion tensors for the 900 MHz ^15^N *R*_2_/*R*_1_ data set. The corresponding data for the full protein is shown in Supplementary Fig. S2. **e** Ellipsoid representation of the diffusion tensors (900 MHz ^15^N *R*_2_/*R*_1_ data) mapped on the crystal structure of MALT1(PCASP-Ig3)_339–719_ with Ig3 and PCASP depicted in red and blue, respectively. The grey part of the structure comprising residues 563–582, including the loop and the α1 helix was excluded from optimization in ROTDIF. The procedures underlying the selection of residues in each domain is presented in the material and method section. The principal axis of the axially symmetric diffusion tensors crosses the poles of the ellipsoids. The diffusion tensor calculated for the full protein is shown in Supplementary Fig. S3.

The diffusion tensor components *D*_||_ and *D*_⊥_ from resampled ROTDIF-optimisations are shown in Fig. 4, separately for PCASP and Ig3 domains calculated using the 900 MHz ^15^N *R*_2_/*R*_1_ data, the 800 MHz ^15^N *R*_2_/*R*_1_ data and the 900 MHz CCR data, respectively. The diffusion tensor data for the full protein is provided in Supplementary Fig. S3. As evident from Fig. 4 and Table 1, the diffusion tensors of the PCASP and Ig3 individual domains are systematically different from each other for all measured data, which clearly indicates a semi-independent movement of these domains relatively to each other in solution^69,70^. The most obvious differences observed in the highest quality *R*_2_/*R*_*1*_ 900 MHz data were still seen in the *R*_2_/*R*_*1*_ 800 MHz and CCR data, demonstrating that all the gathered data are consistent within the experimental error margins. More specifically, the results from the 900 MHz ^15^N *R*_2_/*R*_1_ data revealed an anisotropy ratio (*D*_||_ / *D*_⊥_) of 1.48 and 1.17 for PCASP and the Ig3 domains, respectively. These ratios were 1.43 and 1.28 for the PCASP and Ig3, respectively, when assessing the 800 MHz ^15^N *R*_2_/*R*_1_ data. Finally, the corresponding values were 1.29 and 1.22 for PCASP and Ig3, respectively, when assessing the 900 MHz CCR data. In accordance to the *R*_2_/*R*_1_ values, the effective overall correlation times calculated for the PCASP domain were systematically higher compared to the Ig3 domain (Table 1). Thus, for the 900 MHz data, the *τ*_C_ values were 27.4 ± 1.5 ns and 25.5 ± 1.5 ns for PCASP and Ig3, respectively, while the 800 MHz *τ*_C_ values were 28.3 ± 1.9 ns and 26.8 ± 2.5 ns for PCASP and Ig3, respectively. The CCR-data revealed only a minor difference in correlation times, with values of 28.0 ± 2.8 ns and 27.8 ± 2.1 ns for PCASP and Ig3, respectively. The suggested interdomain dynamics is also well in line with the relatively low anisotropy^70^ obtained from all relaxation data compared to the hydrodynamic prediction^71,72^ using the bead model presenting the protein as a set of spherical beads at the positions of the CA atoms, which gives *D*_||_ / *D*_⊥_ value of *ca* 1.85. Figure 4d, e and Table 1 reveal a difference in the alignment of the tensor symmetry axes for the PCASP and Ig3 domains. While the axis of the PCASP domain is better defined and close to the axis obtained when the diffusion tensor is calculated for the whole protein (Supplementary Fig. S3), the orientation of the axis of Ig3 is different. This apparently unphysical situation^69^ may be explained by the lower anisotropy of the Ig3 domain leading to the poorly defined orientation of its symmetry axis (Fig. 4d). It is also possible that, due to the observed interdomain dynamics, the average orientation of the domains in solution is somewhat different from those defined in the crystal structure, leading to additional systematic error(s) in the symmetry axis orientation^70^.

Although all three datasets agree within the experimental errors and consistently point to differences in the diffusion tensors of the two domains, a trend indicating a progressive reduction of these differences in the 900 MHz, 800 MHz and CCR data warrants a specific discussion. The first factor to consider is the potential impact of the contribution of the conformational exchange *R*_*ex*_ to the *R*_2_ values in the 900 MHz and 800 MHz ^15^N *R*_2_/*R*_1_ data sets, as opposed to the 900 MHz CCR data. The major *R*_*ex*_ contribution can be ruled out, as it would necessitate a longer apparent overall correlation time at 900 MHz than at 800 MHz. Furthermore, the *R*_*ex*_ explanation of the opposite trends in the diffusion tensor values for PCASP and Ig3 requires different *R*_*ex*_ in the two domains, which stands in contrast to the same baseline level of the *R*_ex_/σ_*R*ex_ for the two domains (Fig. 2a). Another potential factor may lie within the ROTDIF procedure, where the diffusion tensor is determined with the assumption of no motions in the nano-second time scale, which may affect the *R*_2_/*R*_1_ data primarily via the *R*_1_ values. Indeed, the tendency for higher *τ*_C_ values in *R*_2_/*R*_1_ analyses in lower magnetic fields has been previously reported for several proteins and attributed to the unaccounted effects of nanosecond motions^73^. However, similarly to the above arguments for the *R*_*ex*_ case, the nano-second motions explanation requires a rather unlikely assumption that these motions are significantly different in the stable/rigid part of the domains used for the tensor calculations. It should be noted that the trend for reduced differences between diffusion tensors in the two domains correlates with the decrease in data quality. Consequently, the primary emphasis in explaining the relative dynamics of PCASP and Ig3 should be based in our opinion only on the highest quality 900 MHz *R*_2_/*R*_1_-data set (Fig. 3a). Finally, it suffices to conclude that the rotation diffusion tensors of PCASP and Ig3 are statistically different (Fig. 4), which can be attributed to the semi-independent rotational diffusion of these domains in MALT1(PCASP-Ig3)_339–719_ in solution.

### Methyl NOE-contacts reveal a pivotal point for the relative motions between PCASP and Ig3

**Fig. 5:**
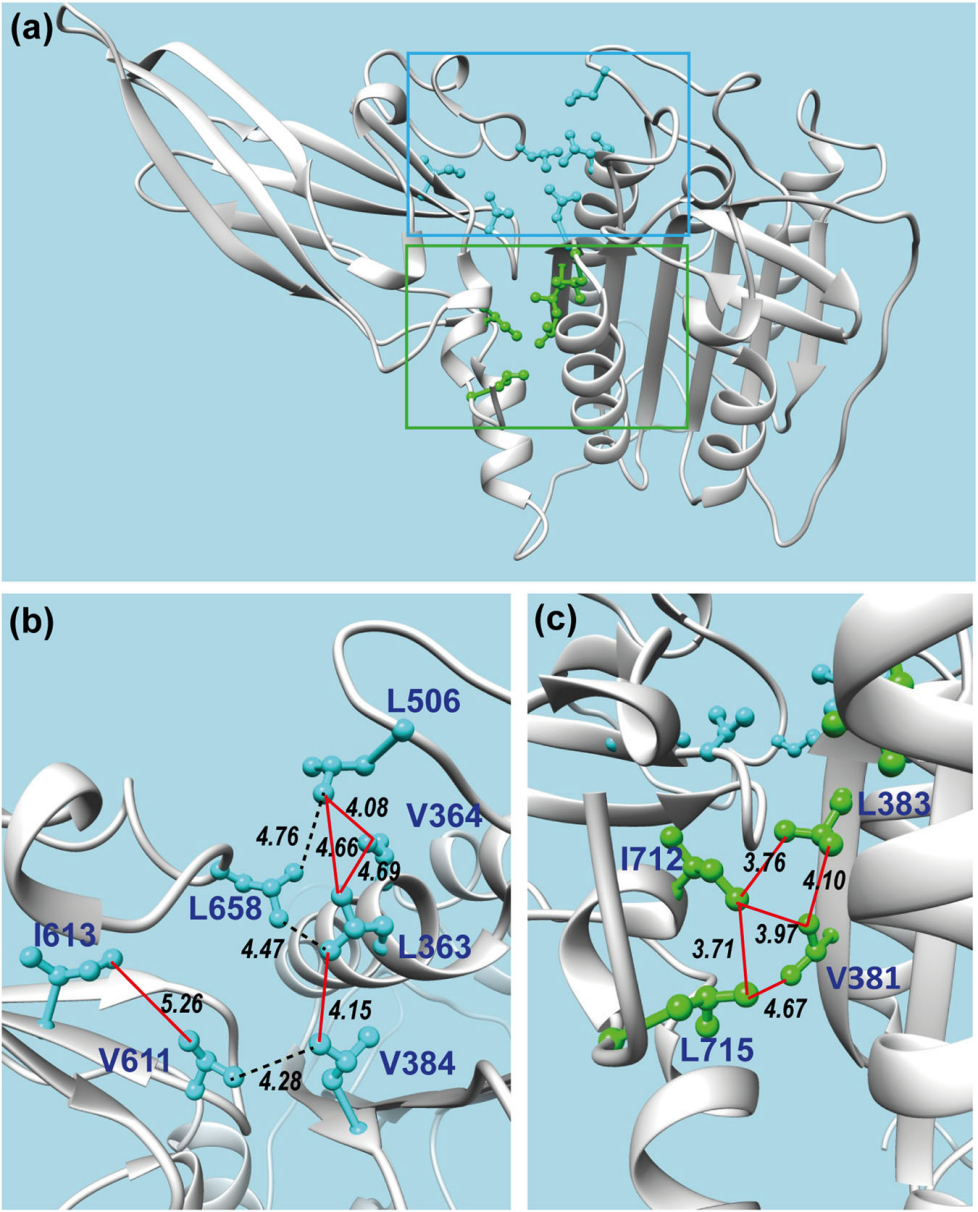
NOE contacts between the side chain methyl groups of MALT1(PCASP-Ig3)_339–719_ at the PCASP and Ig3 domains interface. **a** Overview of the side chains with methyl groups. **b**, **c** Zoomed areas in (**a**) are indicated as blue and green boxes, respectively. Based on the crystal structure of MALT1(PCASP-Ig3)_339–719_ in its apo form, a set of NOEs between the methyl groups were expected in our 4D NOESY experiments. For these, red and dashed black lines mark observed and unobserved NOEs, respectively. Distances (in Å) between the carbons of the methyl groups are indicated. The labels and side chains of the methyl-containing amino acids are also shown.

**Fig. 6:**
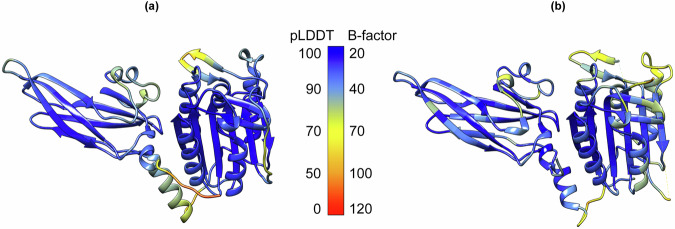
Local confidence of the MALT1(PCASP-Ig3)_339–719_ structures. **a** Per-residue AF confidence scores (pLDDT) calculated as a mean over all the 2000 AF structural models are mapped on the mean structure AF ensemble of MALT1(PCASP-Ig3)_339–719_. The low pLDDT scores 0–50 indicate conformation heterogeneity. **b** B-factor values ranging from 20 – 120 are mapped on the crystal structure of the apo form of MALT1(PCASP-Ig3)_339–719_ (PDB code 3V55).

To assess the semi-independent motion of MALT1(PCASP-Ig3)_339–719_ in its apo form, we examined and analysed the NOE contacts between the CH_3_-CH_3_ protons in the 4D spectrum. Numerous intra and inter domain NOE contacts between the methyl groups could be predicted from the crystal structure of the apo form of MALT1(PCASP-Ig3)_339–719_ (Fig. 5a). The predicted interdomain NOEs (illustrated by a black dashed line in Fig. 5b) are not detected in the upper region part of the domain interface (Blue box in Fig. 5a). Conversely, a chain of NOE intra domain contacts for the same methyl groups is clearly observed (red lines in Fig. 5b). This specific NOE pattern, where contacts exist within the domains but are absent between them, aligns well with our results on the rotational diffusion tensors of the PCASP and Ig3 domains, and the conclusion of their semi-independent motions in solution. On the other hand, a different NOE pattern is also evident in the lower-middle region of the domain interface (Green box in Fig. 5a). Notably, strong NOEs are observed between the methyl groups in both PCASP and Ig3, both within and between these domains (Fig. 5c). This finding suggests that dynamics between these two domains is restricted in this particular patch. Further down at the bottom, the α1 helix followed by the loop connecting the two domains exhibit high structural flexibility as evident from our NMR results (Figs. 2b, 3c, d, 6b), as well as the missing electron densities and the high B-factors observed in the previously determined crystal structure of the apo form of MALT1(PCASP-Ig3)_339–719_ (PDB code 3V55). In summary, our results lead us to conclude that the lower-middle region of MALT1(PCASP-Ig3)_339–719_ may serve as a pivotal point for semi-independent movements of the two domains.

### Dynamics analyses using AlphaFold, NMA, and MD

Although several important static structural snapshots of MALT1 have been obtained through X-ray crystallography studies, an unambiguous understanding of allosteric interactions and their relation(s) to the activity regulation of MALT1 remains challenging. The present study aims to shed light on these complex interdomain relations. Our experimental data revealed unambiguous fast dynamics between PCAP and Ig3 domains, suggesting the existence of multiple conformational substates.

### Quality assessment and dynamics of molecular models of MALT1(PCASP-Ig3)_339–719_ predicted by AlphaFold

We generated 2000 molecular structural models of MALT1(PCASP-Ig3)_339–719_ using AlphaFold. AF assesses its prediction confidence through two main scores; (i) the predicted template modelling score (pTM) (Zhang Y 2004)^59^ and (ii) the per-residue model confidence score (pLDDT)^59,74^. The pLDDT score provides a per-residue estimate of model accuracy, ranging from 0 (low confidence) to 100 (high confidence). Notably, all 2000 structures predicted by AF consistently displayed high pTM scores, ranging from 0.89 – 0.92. As illustrated in Fig. 6a, the pLDDT scores were also high throughout the structures of each created model of MALT1(PCASP-Ig3)_339–719_. However, specific regions of the protein exhibited relatively lower scores as highlighted in yellow in Fig. 6. Recently a direct correlation was established between protein pLDDT and backbone N–H NMR order parameters S^2^ characterising intramolecular dynamics of protein backbone^61^. Accordingly, the regions with low pLDDT scores, such as the α1 helix of the Ig3 domain formed between residues 569 and 583, the loop in the active centre ranging between residues 496 and 509, and the region comprising residues 640–674 in MALT1(PCASP-Ig3)_339–719_, should also have low order parameters S^2^, indicating their high intramolecular dynamics. Importantly, as demonstrated in this study (Fig. 3c, d), the same regions in MALT1(PCASP-Ig3)_339–719_ displayed low values for the local correlation times *τ*_C,i_ and the ^15^N-(^1^H)NOEs values, thus experimentally indicating that the dynamics are within the ps - ns time scale. Furthermore, the B-factors derived from the crystal structure of MALT1(PCASP-Ig3)_339–719_ (Fig. 6b) demonstrated notable correlation with the pLDDT scores. The lowest B-factors aligned well with conformational heterogeneity, specifically in the third loop within the active centre (residues 496–509) and the region comprising residues 640–674. The α1 helix in Ig3 appeared somewhat better defined and rigid in the crystal structure, which does not correlate with our relaxation and AF data. This may be due to the stabilisation of this region by crystal packing. Indeed, the same region is poorly defined in other crystal structures of MALT1(PCASP-Ig3)_339–719_ with different space groups (PDB codes 3UOA and 3UO8).

### PCA analysis of the AlphaFold ensemble of structures

**Fig. 7:**
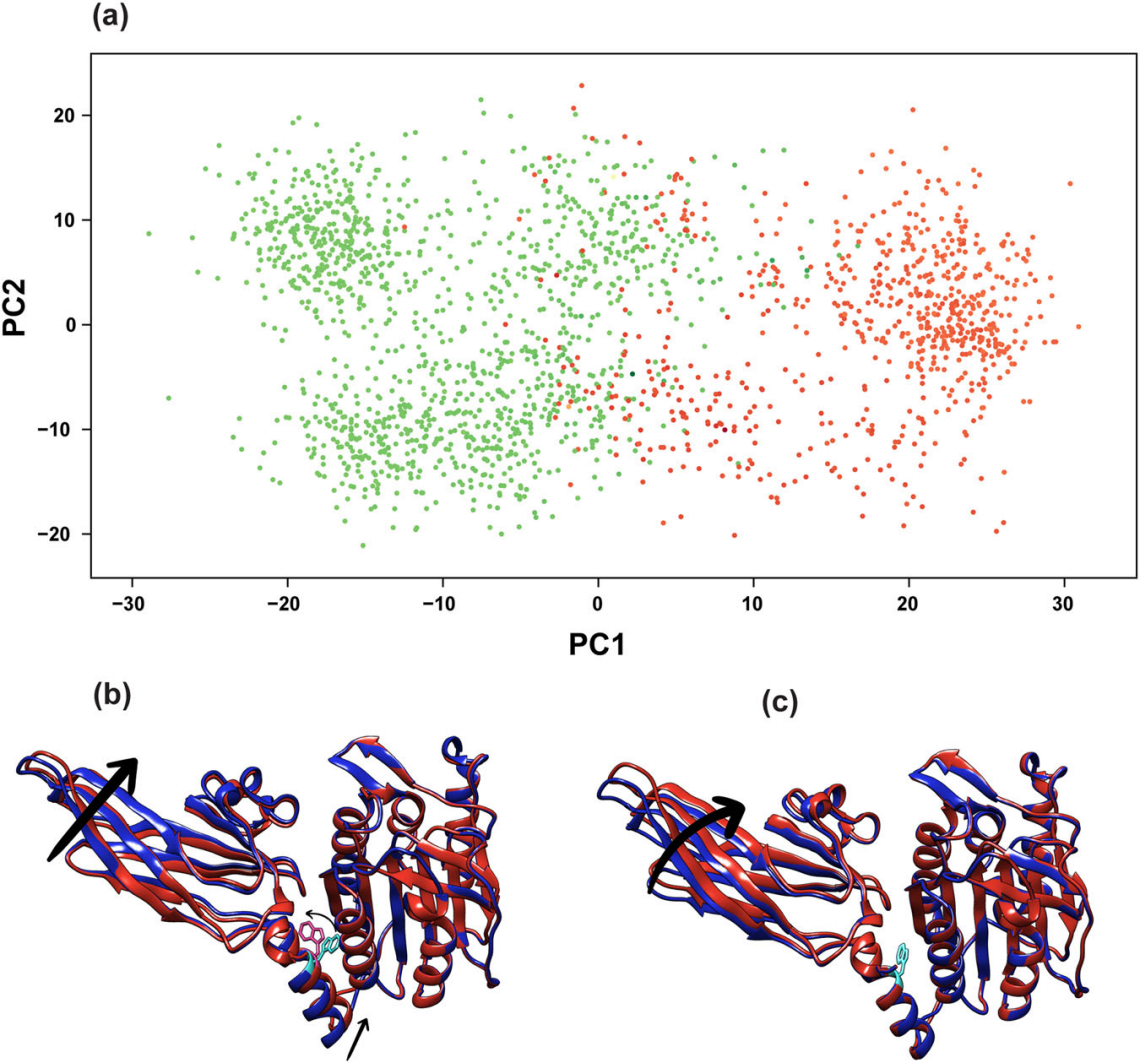
Principal component analysis performed on backbone atoms (PCAa) of MALT1(PCASP-Ig3)_339–719_ models generated using AlphaFold indicates semi-independent movements of both domains pivoting around the tryptophan residue W580. **a** Scatter plots depict 2000 distinct conformations in the PC1 and PC2 frames, revealing the conformational heterogeneity within AF models. Conformations with outward-facing and inward-facing W580 are represented in green and orange, respectively. **b**, **c** The superposition of the two structures shown in red and blue captures the two extremes of the structural variations in the conformational ensemble along the PC1 (**b**) and PC2 (**c**) components, respectively. Arrows indicate the direction of coordinated variation between Ig3, the α1 helix, and residue W580 in MALT1(PCASP-Ig3)_339–719_.

To place the predictions by AF of conformational variance of MALT1(PCASP-Ig3)_339–719_ in context with the experimentally obtained dynamic data within this study, we utilised principal component analysis (PCA). PCA effectively reduces the multidimensional space to a smaller, representative space that emphasises the primary conformational heterogeneity and motions. We initially conducted PCA (denoted as PCAa) on all 2000 structures predicted by AF encompassing the backbone N, Cα, C, and O atoms of protein residues 344 to 717. The first two principal components (PC1, and PC2) captured 48% and 17% of the structural variations among the AF models, respectively. The PC1/PC2 scores for individual structures from the ensemble shown in Fig. 7a, revealed that the predicted conformations do not follow a simple interpolation between two end states, but instead, form a conformationally diverse ensemble of models. To reveal heterogeneity and cooperativity within the conformational ensembles of MALT1(PCASP-Ig3)_339–719_ captured by the PC1 and PC2 components in PCAa, we created a visual representation of the PCA loadings by superposing two structures representing the beginning and the end of the structural variations identified in PC1 and PC2, respectively (Fig. 7b, c). Following the direction of coordinated variation between Ig3 and the α1 helix of MALT1(PCASP-Ig3)_339–719_ across the PC1 and PC2 components, it became evident that conformational changes in Ig3 are highly intricate (Fig. 7b). Notably, a strong correlation unfolded within the PC1 component including bending of the Ig3 domain and displacement of the α1 helix between the PCASP and Ig3 domains (Fig. 7b). These observations align well with our NMR data, highlighting the semi-independent movements of Ig3 and PCASP, both pivoting around a crucial point in the lower-middle region of their interface. It should also be noted that the PC2 component indicates that the Ig3 domain partially rotates around an internal axis (Fig. 7c).

An intriguing yet unexpected discovery in our PCAa analysis of the AF dataset’s PC1 component is the conformational flip of the aromatic ring of residue W580 over the χ2 angle. Conformations with outward-facing and inward-facing orientations of residue W580 were identified, coloured in green and orange respectively, and clustered into two distinct families (Fig. 7a, c). Notably, the conformational flip observed for W580 is strongly correlated with the PC1 score and hence with the conformation of the α1 helix.

**Fig. 8:**
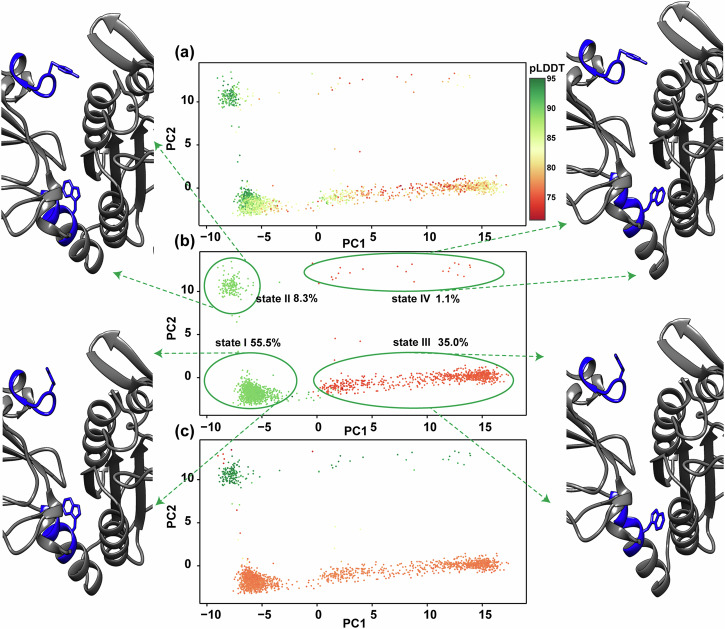
PCAb analysis of the MALT1(PCASP-Ig3)_339–719_ conformational ensemble predicted by AF identifies four distinct clusters. The alternative conformations of MALT1(PCASP-Ig3)_339–719_ modelled by AF are depicted in (**a**) (**b**) and (**c**) featuring dimensionality reduction through the principal component analysis (PCAb), and illustrating the clustering of predicted conformations. In (**a**), the model confidence is colour-coded using the pLDDT score for residue W580 in the range 71.3–95.1 with higher values indicated in green, signifying greater model confidence. As illustrated in panel (**b**), dimensionality reduction revealed four distinct states (I–IV) based on the position of the aromatic rings of residues W580 and Y657, surrounded by green curves with estimated populations. Structural changes between the AF clusters are highlighted, and representative structures for each state are presented. The different orientations of the aromatic rings of W580 and Y657 are depicted in orange. Additionally, PCA models are coloured in (**b**) according to the χ2 angle score of W580, ranging from ~ 80° (green) to ~−100° (orange). This clustering corresponds to either outward-facing or inward-facing W580 with respect to PCASP, respectively. In (**c**), PCA models are coloured based on the χ1 angle score of residue Y657, ranging from −93° (orange) to 57° (green). This colouring separates the inward-facing and outward-facing orientations of the side chain of residue Y657, respectively.

Next, to further explore the potential structural rearrangements within the conformational ensembles of the Ig3 domain in the AF molecular models, induced by variations in the conformation of the aromatic ring of residue W580, we performed an additional PCA analysis, denoted as PCAb (PC1 74% and PC2 13%). This analysis presented in Fig. 8 focused on the two short stretches of residues 579–584 and 652–659 in MALT1(PCASP-Ig3)_339–719_, encompassing both aromatic rings of W580 and Y657. The primary and pivotal observation derived from these analyses was that when the aromatic ring of W580 flips out of the allosteric pocket, taking an outward-facing orientation with a χ2 angle of~80°, the aromatic ring of residue Y657 can adopt either of two χ1 angles, –93° or 57°. The 25 Å distance between the Cα-atoms of W580 and Y657 is large. These values correspond to conformational states I and II (Fig. 8b). In contrast, when the aromatic ring of W580 flips towards the allosteric pocket with a χ2 angle of about −100°, the aromatic ring of Y657 consistently maintains a dominant position with a χ1 value of –93°, turned away from the third loop of the active site. This corresponds to conformational states III and IV, as presented in Fig. 8b. The identified cluster populations are distributed as 55.5%, 8.3%, 35.0%, and 1.1% for states I, II, III, and IV, respectively (Fig. 8b). According to our findings based on the AF prediction models, an equilibrium could exist between the two main conformations taken by the aromatic residue W580. Nevertheless, models with the highest confidence, according to the pLDDT score of W580, were predominantly found in states I and II (Fig. 8a), which led us to the conclusion that, based on AF predictions, a conformational ensemble with the side chain of W580 in the outward orientation is more likely. Additionally, we repeated the sampling of predictions for MALT1(PCASP-Ig3)_339–719_ using alternative statistical methods RoseTTAFold and ESMFold, which both provided results that closely resemble those obtained with the AF models (Supplementary Fig. S4). This is illustrated by the distribution of the pLDDT scores depicted on the structures, and by the predicted conformations adopted by the side chains of the aromatic residues W580 and Y657. Notably, similar to the most populated AF conformation, both RoseTTAFold and ESMFold structures have the side chain of W580 taking an outward-facing orientation.

### PCA analysis of MD and NMA structures

To investigate potential transitions between the alternative conformations of MALT1(PCASP-Ig3)_339–719_, revealed in the AF ensemble of structures, we employed two computational approaches for modelling molecular dynamics (Supplementary Fig. S5); an all-atom Molecular Dynamics with explicit solvent and Normal Mode Analysis (NMA). The methods explore local and global minima of conformational ensembles, depending on the force field used in the calculations. Supplementary Fig. S5a–c display the superpositions of two structures captured at the opposite ends of a structural span in all the conformational ensembles obtained from the first three NMA modes. Additionally, Supplementary Fig. S5d, e depict the loadings of the PC1 and PC2 components of a PCA performed on the 2000 structures extracted from a 2 μs MD trajectory. Visual inspection of Supplementary Fig. S5 highlights similarities between the conformational diversity exhibited by the Ig3 domain in the MD and NMA calculations, which is also well in line with the analyses of the AF conformational ensembles described here above. It is noteworthy that both NMA and MD do not display any notable diversity in the orientations of the α1 helix in MALT1(PCASP-Ig3)_339–719_. Accordingly, no coupling was observed between the positioning of the Ig3 domain and the orientation of the α1 helix. Furthermore, during the full MD trajectory, we were unable to detect any transition from the inward orientation of the aromatic ring of W580, as identified in the initial structure, to the outward orientation preferred by AF, ESMFold2 and RoseTTAFold2. This may be explained by the intrinsically local conformational sampling in NMA and possibly insufficiently long runs of the MD traces to identify rare conformational transitions.

### Analysis of the free energy

**Fig. 9:**
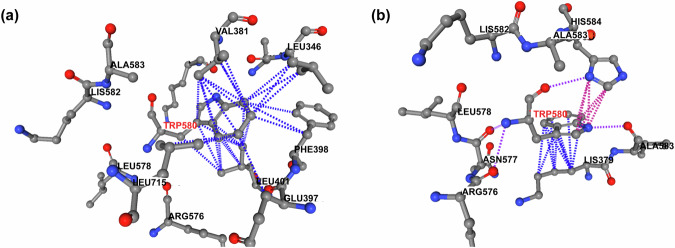
Hydrophobic and aromatic contacts between the aromatic ring of W580 of MALT1(PCASP-Ig3)_339–719_ at the interface of the PCASP and Ig3 domains. **a** depicts the hydrophobic pocket observed in the X-ray structure (PDB code 3V55) of the apo form where the aromatic ring of W580 faces inward into the allosteric site; (**b**) shows interactions observed in the X-ray structure of the complex MALT1(PCASP-Ig3)_339–719_ (PDB code 7AK1) with an inhibitor in the allosteric site, where aromatic ring of W580 faces outward, i.e. away from the allosteric site. The hydrophobic contacts are shown by blue dotted lines, and aromatic-aromatic interactions are indicated by pink dotted lines.

The effects of the orientation of W580 on the free energy were analysed using the PermPS method^75^. We estimated ΔΔG induced by a single mutation W580A in several crystal and AF structures (Supplementary Table S4), representing the outward (states I and II in Fig. 8b) and inward (states III and IV in Fig. 8b) orientations of W580. It should be noted that ligands, if any in the crystal structures, were not considered in the ΔΔG calculations. For all structures, the W580A mutation was destabilizing with a positive ΔΔG, and an average value 1.85 ± 0.13 and 1.18 ± 0.17 kcal/mol for the inward and outward states, respectively. The former value is close to the predicted effect of the naturally occurring destabilisation mutations W580S (ΔΔG = 1.78 kcal/mol)^37^ and W580R (ΔΔG = 1.81 kcal/mol)^76^. Figure 9 shows a network of favourable interactions that stabilize the tryptophan ring in both orientations and are absent in the mutated W580A variants. While in the inward configuration, the tryptophan ring of W580 is involved in multiple hydrophobic interactions, the outward position is stabilized by aromatic interactions with histidine H584. The comparison of the free energy in different MALT1(PCASP-Ig3)_339–719_ structures in the apo form, *i.e*. those with alternative W580 orientations, is challenging and requires an extensive molecular dynamic modelling^77^. However, an estimate of the free energy contribution limited to the direct W580 interactions can be obtained by comparing ΔΔG values for the W580A mutants, which gives on average 0.67 ± 0.2 kcal/mol lower energy for the inward configuration and corroborates with the dominance of the inward configuration reported our experiments.

## Discussion

The exploration of protein dynamics is essential for gaining insights into biological processes at an atomic and molecular level. Despite the extensive biochemical and structural research conducted on MALT1 over the last decade, an investigation of its dynamics has not been performed. In this study, we focused on the dynamics of MALT1(PCASP-Ig3)_339–719_ in order to assess and better understand the complex interplay between the monomeric and dimeric states, domain interactions, conformational space sampling. Our investigation began by addressing whether the apo form of MALT1(PCASP-Ig3)_339–719_ in solution exists predominantly as a monomer or as an equilibrium between monomeric and dimeric states. Using classical gel-filtration, we isolated the monomer form of MALT1(PCASP-Ig3)_339–719_, which was subsequently employed in our solution-state NMR experiments. Our NMR results and analyses confirmed that MALT1(PCASP-Ig3)_339–719_ remains primarily monomeric in our experimental settings. In cells, induced proximity of the PCASP-Ig3 in the CBM complex is required to activate MALT1 and thus the question remains if the proximity may be sufficient for inducing dimerization.

According to the crystal structure of the apo form of MALT1(PCASP-Ig3)_339–719_, the Ig3 domain interacts tightly with the PCASP domain through the formation of several hydrophobic contacts, which are affected upon substrate binding. Residue W580 plays a crucial role in maintaining this interaction, contributing to a stable protein fold by binding to a hydrophobic pocket in PCASP, often called the allosteric pocket, and bridging it to Ig3. In contrast, our present NMR-based dynamic study of the monomeric form of MALT1(PCASP-Ig3)_339–719_ reveals a captivating complexity in domain interactions. Instead of forming a tight complex or being fully independent, the PCASP and Ig3 domains exhibit a dynamic, semi-independent movement around a pivotal point. Interdomain motion is often implicated in allosteric pathways^44,78^.

Utilising AF and PCA, our study provides insights into the conformational space sampling of the PCASP and Ig3 domains detected by our NMR relaxation experiments. The bend and twist movement of the Ig3 domain (Fig. 7) seems to be coupled to the orientation taken by the α1 helix connecting the PCASP and the Ig3 domains. The AF structural ensemble that we obtained in this study is generally well in line with the structural variations observed in the multiple crystal structures of MALT1(PCASP-Ig3)_339–719_ that have been previously determined both in its apo- and its ligand-bound forms, including the span of relative orientations of PCASP and Ig3, as well as the multiple orientations taken by the α1 helix of the Ig3 domain. As a control for the results predicted by AF, we produced structural models of MALT1(PCASP-Ig3)_339–719_ using the two alternative AI-driven approaches ESMFold2^79^ and RoseTTAFold2^80^ that, similarly to AF, exploit multiple sequence alignments (MSA). Despite their functional similarity, it is crucial to note conceptual and architectural differences in these three programmes, as previously highlighted^80,81^. Our results revealed that both RoseTTAFold2 and ESMFold2 successfully reproduced the structural models obtained by AF. The quality of the predicted models was assessed using the per-residue RoseTTAFold2 and ESMFold2 confidence scores (pLDDT) (Supplementary Fig. S5), which show clear similarities with the AF pLDDT scores (Fig. 6) and more specifically in the connecting α1 helix of Ig3. Notably, both RoseTTAFold2 and ESMFold2 models show the outward orientation of the W580 side chain, which is also the most dominant conformation in the AF conformational ensemble analysis (Fig. 8).

A new intriguing aspect emerged in the detailed analysis of the AF ensemble obtained for the apo form of MALT1(PCASP-Ig3)_339–719_. Here, the aromatic ring of residue W580 adopted both orientations found in previously determined crystal structures, with an inward conformation, where the side chain ring resides within the allosteric pocket, and the outward-projecting conformation. However, somewhat surprisingly, the most represented of the two conformations taken by W580 was the outward aromatic ring conformation (Fig. 8b), which was previously found in crystal structures of MALT1(PCASP-Ig3)_339–719_ in complex with allosteric ligands occupying the allosteric site. Conversely, in the apo form of MALT1(PCASP-Ig3)_339–719_, W580 adopts the inward-looking orientation and occupies the allosteric pocket. Based on static crystallographic data, it is commonly accepted that the aromatic ring of W580 is pushed out from its position in the hydrophobic allosteric groove into a solvent-exposed environment upon binding of an allosteric inhibitor by an induced fit mechanism, followed by a substantial displacement of the α1 helix of Ig3^36^.

Our current NMR analysis of the apo form of MALT1(PCASP-Ig3)_339–719_ allowed us to unambiguously identify in solution only one of the AF-predicted forms where W508 resides in the allosteric pocket. To determine the dominant form of W580 in the apo form of MALT1(PCASP-Ig3)_339–719_ in solution, we utilised the fact that in the conformation where the aromatic ring W580 is in the inward orientation and resides in the allosteric pocket (Fig. 8b, **states III** and **IV**), one of the methyl groups in each of the V381 and V401 residues are positioned almost exactly above and below, respectively, relative to the aromatic ring of W580. In this configuration, the protons of these methyl groups experience a large upfield chemical shift due to the aromatic ring effect of the side chain of W580^82^. Indeed, chemical shifts of the protons of V381Cγ2 and L401Cδ1 were observed in the ^1^H-^13^C HSQC spectrum of MALT1(PCASP-Ig3)_339–719_ at very high upfield positions, −0.577 and −0.505 ppm, respectively. This contrasts with V381Cγ1 and L401Cδ2, where the shielding effect of the aromatic ring is minimal, at 0.467 and 0.414 ppm, respectively. The result on the methyl chemical shift points to the inward orientation of the W580 aromatic ring as the dominant configuration for the apo form of MALT1(PCASP-Ig3)_339–719_ in our experimental conditions. This means that the alternative protein conformation with the outward W580 orientation, if present at all, may exist only as a minor low populated state, which would be corroborated by the conformational exchange observed in the helix α1 (Fig. 2) and reported in our previous ^15^N relaxation dispersion study^83^. Results of free energy estimates are also in line with the dominance of the inward configuration, while not excluding the alternative outward form. Surprisingly, this orientation of W580 corresponds to the least populated cluster in the AF ensemble. Does this mean that the AF, ESMFold2, and RoseTTAFold2 algorithms, which are based on statistics and/or artificial intelligence (AI) combined with genome sequence analysis, provide erroneous molecular models for MALT1(PCASP-Ig3)_339–719_ by preferring the conformation of the side chain of W580 localized outside the allosteric hydrophobic grove?

There is credible criticism in recent literature^84–86^ regarding the ability of AF to predict correct partitioning in structural ensembles. It was argued that one can only give full credence to conformations that have been experimentally validated and dismiss those that have not undergone validation. Consequently, although the outward conformation of the side chain of W580 has been observed in crystal structures of ligated MALT1(PCASP-Ig3)_339–719_, the dominance of this ensemble in the apo form of the protein may be seen as a bias in the AF model. On the other hand, despite acknowledging limitations in AF conformation predictions, a number of interesting results were recently presented^63^ that encourage further studies on AF structural ensembles. It is essential to acknowledge that the AF algorithm lacks specific information about the environment conditions for the conducted experiments. One can argue that modelling using statistics from three-dimensional structures found in the Protein Data Bank (PDB) and amino acid sequences (*i.e*. MSA) could instead reflect the likeliest PDB structural motives and cellular context and functional relevance in evolution. Specifically, while distance constraints derived from MSA reflect evolutionary and thus functionally important structures, statistics derived from PDB structures reflect likely structural features and contexts, *e.g*. interactions with other molecules, found in the PDB. To this end, it is not surprising that all our AF molecular models display PCASP in its functional enzymatically active configuration, although this state was crystallised only with ligands bound at the active site^27^, but not for the apo-form of the protein. Furthermore, even when in an inactive resting state, MALT1 can be activated by a substrate or changing environmental conditions^27^, such as in the presence of kosmotropic salts^24^. This underscores the importance of recognizing the potential for shifts in populations within the conformational ensemble in solution. Both AF-predicted conformations of W580 may have a sizable representation in solution, even though the facing-outward conformation of W580 was not observed and thus low populated under the specific conditions of our experiments. Altogether, our results allow us to extend the structural perspectives that emerged from previous X-ray studies of MALT1(PCASP-Ig3)_339–719_, towards acknowledging the existence of diverse conformational ensembles in solution and underscoring the necessity of considering solution dynamics when designing function modulating ligands for MALT1. More specifically, based on our present results, we argue that binding of an allosteric drug involves a conformational selection that repartitions the conformational ensemble with stabilization of the flipped-out configuration rather than causing W580 to switch^37^ its conformational positions to vacate the groove space for the inhibitor. Finally, and perhaps the most disputable and intriguing possibility would be to accept that the AF partitioning of the conformational states reflects a real albeit hitherto unknown environment situation in vivo. Applied to the MALT1(PCASP-Ig3)_339–719_ system, AF strongly prefers molecular models where W580 turns away from the hydrophobic groove (Fig. 8a). This conformation is usually observed in vitro at experimental conditions when the proteolytic activity is inhibited by an allosteric ligand. Noting that the AF algorithm runs without the structural templates and lacks knowledge about MALT1(PCASP-Ig3)_339–719_ crystal structures with specific unnatural inhibitors, we could hypothesise that the predicted AF dominant conformation prevails in vivo, may be stabilized by an as-yet-unknown natural inhibitor/factor. This may be used by the cell to limit the function of MALT1 solely to its scaffolding role. Indeed, recent in vitro study involving the mutation of W580 to a serine^37^ demonstrated that upon binding allosteric inhibitors to MALT1-W580S, the protein scaffold functions can be restored by increasing protein stability in the cell through the rescue of canonical NF-κB and c-Jun N-terminal kinase (JNK) signalling. Along the same line, similar to the W580S and W580R mutants^76^, the outward configuration of W580 may be inherently prone to proteasomal degradation and thus require stabilization by the hypothesized natural inhibitor. The PCA analysis (PCAb) of AF molecular models conducted here on the two short stretches of residues 579–584 and 652–659 in MALT1(PCASP-Ig3)_339–719_ allowed us to examine further potential structural rearrangements within the conformational ensemble of the Ig3 domain. These regions encompass both the aromatic rings of residues W580 and Y657. Here, the key observation is that when the aromatic ring of W580 faces outward from the allosteric pocket, the aromatic ring of Y657 can adopt either of two χ1 angles, ranging between −93° and 57°, representing conformational states I and II (Fig. 8b). Conversely, when the aromatic ring of W580 is oriented towards the allosteric pocket, the aromatic ring of Y657 consistently maintains a dominant position with a χ1 value of −93°, turned away from the third loop of the active site. This corresponds to conformational states III and IV (Fig. 8b).

It is also important to highlight a recently reported alternative allosteric pathway for restoring and regulation of the protease function of MALT1 through monoubiquitination^35^. The study suggests that Ubq attachment to residue K644 in the Ig3 domain induces conformational changes in the Ig3-protease interface, ultimately enhancing MALT1 activation while pointing to the importance of Y657 as a key mediator in signalling between Ig3 and protease domains. It is noteworthy that in the AF-predicted state II (Fig. 8b), the aromatic Y657 potentially impacts on hydrophobic inter-domain interactions, resembling the proposed structural alteration seemingly provoked by covalent attachment of Ubq to K644^35^. Additionally, cluster II (Fig. 8b) features orientation of the Y657 aromatic ring as in the crystal structure where the protein binds the substrate analogue z-VRPR-fmk. Based on this similarity, we argue that the conformational equilibrium between states I and II for MALT1 may shift through monoubiquitination, leading to activation of MALT1 protease function. It is noteworthy that only states III and IV, characterized by the inward configuration of W580, are observed in NMR experiments of MALT1(PCASP-Ig3)_339–719_ in solution. Furthermore, the active form IV, in which residue Y657 interacts with the active site, has a cluster population of only 1.1% as predicted by AF. Altogether, these observations may explain the lower activity of the monomer in solution.

Next, we made an additional effort to understand the movements responsible for potential transitions between different conformations of the aromatic amino acids W580 and Y657 in MALT1(PCASP-Ig3)_339–719_, identified in the AF models. Using a structure from the state III family as a starting point (Fig. 8), we conducted long-sampling conformation trajectories through a 3 μs MD simulation. This MD simulation successfully reproduced the substantial flexibility of the Ig3 domain and predicted the conformational flips of the aromatic ring of Y657, as observed in AF modelling. However, the most crucial finding was that MD simulations did not indicate any conformational changes for the side chain of W580 in the allosteric pocket, as seen in the State I or II families from AF modelling (Fig. 8b). Similar results were obtained in Normal Mode Analysis. Additionally, a PCA of the 2000 structural ensembles extracted from the MD trajectory shows neither diversity in the conformational ensemble between the α1-helix and the PCASP domain of MALT1(PCASP-Ig3)_339–719,_ nor the conformational flips of the aromatic ring W580 out of the allosteric pocket. This is not surprising since PCA analysis of the AF ensembles indicated coupling of these two movements. Two main factors may explain the divergence between the MD and AF results. First, the MD force field and AF modelling are not precise. Secondly, the transition from the global energy minima of the W580 conformation likely requires an even more extended MD trajectory time. This is supported by the measured higher value of the exchange rate Rex in the interdomain linker, including in the α1-helix, indicating the presence of slow exchange. To further investigate the possibility of slow exchange or transitions between W580 outward and inward orientations, future measurements of relaxation dispersion on the aromatic ring of W580 and methyl protons of V381 and L401 residues are needed. These measurements could provide insights into the dynamics and transitions of W580 between different conformations.

We have performed the first comprehensive NMR study of MALT1 dynamic behaviour in solution. Measurements and analyses of the ^15^N-backbone *R*_1_- and *R*_2_-relaxation, as well as the CCR rates of MALT1(PCASP-Ig3)_339–719_ and the mutated E549A variant, revealed a complex picture of motions including overall rotational diffusion, inter-domain motion and local internal dynamics. Our NMR relaxation results confirm that MALT1(PCASP-Ig3)_339–719_ is a monomer in our experimental conditions. Furthermore, the PCASP and Ig3 domains do not form a tight complex but instead exhibit semi-independent movements around a pivotal point. To model the movement between PCASP and Ig3 domains and characterise the conformational ensemble for the MALT1(PCASP-Ig3)_339–719_, we employed AlphaFold, followed by a mechanistic rationalisation of the conformational space using principal component analysis. The AF modelling unveils strong correlations between the interdomain movements, bending of the Ig3 domain, and the displacement of the α1 helix located between the PCASP and Ig3 domains. Furthermore, displacements of the α1 helix in the AlphaFold ensemble of structures are highly correlated with the orientation of the aromatic ring of W580 located in the interdomain pivot region. Then, the orientation of W580 is a marker of the accessible states of the Y657 located 25 Å away in the interdomain interface close to the PCASP catalytic site, which is most probably consequential for the allosteric regulation of the protein catalytic activity.

According to the AF structural ensemble, an equilibrium could exist, probably even in the apo form of MALT1(PCASP-Ig3)_339–719_, between conformations with two orientations of the aromatic ring in W580, notably including the outwardly facing conformation previously experimentally observed in MALT1(PCASP-Ig3)_339–719_ complexes with the allosteric inhibitors occupying the “native” position of the W580 side chain. The outward conformations were also found in models provided by alternative AI-based methods RoseTTAFold and ESMFold. To further explore the putative potential transitions between the alternative conformations of MALT1(PCASP-Ig3)_339–719_ predicted by AF models, we performed a 2 μs MD simulation. The observed conformational diversity in the Ig3 domain aligns with data obtained in AF models, yet the transition of the aromatic ring of W580, as predicted in AF, is not detected within the MD trajectory. Finally, we discuss the validity of AF-predicted conformational ensembles and more specifically the relevance and possible implications in the context of complex cellular environment of the preference of the outward facing W580 conformation preferred by AF, RoseTTAFold, and ESMFold without presence of the allosteric inhibitor.

In summary, our study considerably extends the understanding of MALT1 dynamics and may shed light on structural details and its functional and regulation. The revelations regarding domain interactions, conformational space sampling, and apo form dynamics pave the way for further exploration of MALT1’s role in cellular processes and the development of targeted therapeutic interventions. As we delve deeper into the intricate realm of protein dynamics, MALT1 emerges as a captivating case study, illustrating the dynamic nature of molecular systems in biological processes.

## Materials and methods

### Theoretical background

The theory of nuclear magnetic relaxation is well described^67,72,87,88^. In the two-spin ^1^H-^15^N system, the measured relaxation parameters *R*_1_, *R*_2_, the ^15^N–(^1^H) nuclear Overhauser effect (NOE), and the cross-correlated relaxation ${\eta }_{{xy}}$ are connected to the molecular dynamics via the spectral density function *J(ω)*:

1a $${R}_{1}=\frac{1}{4}{d}^{2}\left[J\left({\omega }_{H}-{\omega }_{N}\right)+3J\left({\omega }_{N}\right)+6J\left({\omega }_{H}+{\omega }_{N}\right)\right]+{c}^{2}J\left({\omega }_{N}\right)$$

1b $${R}_{2}= 	\, \frac{1}{8}{d}^{2}\left[4J\left(0\right)+J\left({\omega }_{H}-{\omega }_{N}\right)+3J\left({\omega }_{N}\right)+6J\left({\omega }_{H}\right)+6J\left({\omega }_{H}+{\omega }_{N}\right)\right] \\ 	 +{\frac{1}{6}c}^{2}[4J\left(0\right)+3J\left({\omega }_{N}\right)]+{R}_{{ex}}$$

1c $${NOE}=\frac{{I}_{{sat}}}{{I}_{{ref}}}=1+\frac{1}{4{R}_{1}}{d}^{2}\left(\frac{{\gamma }_{H}}{{\gamma }_{N}}\right)\left[6J\left({\omega }_{H}+{\omega }_{N}\right)-J\left({\omega }_{H}-{\omega }_{N}\right)\right]$$

1d $${\eta }_{{xy}}={p\delta }_{N}\left[4J\left(0\right)+3J\left({\omega }_{N}\right)\right](3{cos }^{2}\theta -1)$$

where $d=\left(\right.{\mu }_{0}h{\gamma }_{N}{\gamma }_{H}/({8\pi }^{2})\cdot {{\langle }}1/{r}_{{NH}}^{3}{{\rangle }}$; ${\mu }_{0}=4\pi \times {10}^{-7}H{m}^{-1}$; $h=6.62607\times {10}^{-34}$ Js; ${\gamma }_{N}=-27.116\times {10}^{6}{rad}{s}^{-1}{T}^{-1}$; *r*_*NH*_ = 1.02 Å; $p=\frac{{\mu }_{0}{\gamma }_{H}{\gamma }_{H}h}{16{\pi }^{2}\surd 2{r}_{{NH}}^{3}}$; ${\delta }_{N}=\frac{{\gamma }_{N}{B}_{0}\varDelta \sigma }{3\surd 2}$; $c=\varDelta \sigma \cdot {\omega }_{N}/\surd 3$; $\varDelta \sigma =172\,{ppm}$ is the ^15^N chemical shielding anisotropy; $\theta =17^\circ$ is the angle between the ^15^N − H vector and the unique principal axis of the ^15^N chemical shift tensor; and ${R}_{{ex}}$ is a contribution to ${R}_{2}$ due to conformational exchange in the slow μs-ms time scale. The most simplified model of the spectral density function corresponds to an isotropic molecular rotational diffusion in solution and does not account for the intramolecular motions:

2a $$J\left(\omega \right)=\frac{2}{5}\left(\frac{\,{\tau }_{c}}{1+{\left(\omega {\tau }_{c}\right)}^{2}}\right)$$

where *τ*_C_ is the global molecular rotational correlation time.

In the case of anisotropic rotational diffusion, there is dependence of the spin-relaxation of a given ^15^N nucleus on the orientation of the NH-bond in the molecular coordinate frame, hence the rotational diffusion has directional dependence. The anisotropic model is represented as a sum of five terms:

2b $$J\left(\omega \right)=\frac{2}{5}{\sum }_{i=1}^{5}{A}_{i}\left\{\frac{{\tau }_{c,i}}{1+{\left(\omega {\tau }_{c,i}\right)}^{2}}\right\}$$

where correlation times ${\tau }_{c,i}$ and the corresponding weights *A*_i_ depend on parameters of the rotational diffusion tensor ***D*** and the orientation of the H-N vector relative to the tensor principal axes. Tensor ***D*** is defined by six independent values including its principal components ${D}_{i}$ (*i* = *x, y, z*) and the Euler angles *α, β, γ* that characterise the directions of its principal axes in the reference molecular frame.

The effective overall rotational correlation time is computed according to:

3 $${\tau }_{C}=1/2{tr}(D)=1/2({D}_{x}+{D}_{y}{+D}_{z})$$

For the axially symmetric rotational diffusion Eq. (2b) simplifies by reducing from five to three terms^68,72^ and we can define *D*_||_ ≡ *D*_Z_ and *D*_⊥_ ≡ *D*_X_ = *D*_Y_, and the anisotropy ζ of the diffusion tensors as ζ = *D*_||_ / *D*_⊥_.

When the orientations of the HN vectors are known from the protein 3D structure, the tensor parameters can be calculated using experimental *R*_2_/*R*_1_-ratios for multiple HN vectors^70,72^. The *R*_2_/*R*_1_ method was successfully validated for protein systems up to 25 kDa^67^. For larger systems, the TRACT approach, (TROSY for Rotational Correlation Times)^89^ was proposed, which uses measurements of rates of the cross correlated relaxation$\,{\eta }_{{xy}}$. The TRACT approach makes use of the relaxation interference between the two dominating relaxation mechanisms: the dipole-dipole interaction between amide ^15^N and its directly attached proton, and the amide ^15^N chemical shielding anisotropy (CSA). For ^15^N nuclei attached to a proton in the *β* spin state, the sum of the dipolar and CSA relaxations is smaller than for ^15^N nuclei attached to a proton in the *α* spin state, and thus the two types of ^15^N nuclei relax at very different rates. While experimental ${\eta }_{{xy}}$ are more susceptible to the impact of internal motions compared to *R*_2_/*R*_1_ ratios, it is worth noting that the cross-correlated relaxation is immune to the conformational exchange in the microsecond-millisecond time scale.

The relationship between *η*_xy_ and the effective residue-specific rotational correlation time *τ*_C_ is provided by the equation:

4 $${\tau }_{C}=5\,{\eta }_{{xy}}/({8S}^{2}p{\delta }_{N}(3\,{cos }^{2}\theta -1))$$

where ${S}^{2}$ is the model free backbone N–H order parameter allowing correction for the fast picosecond motions of the HN vector in the molecular coordinate frame. The above equation is a simplified equation from^90^, where in the expression for ${\eta }_{{xy}}$ we kept only the terms proportional to $J\left(0\right)$. We estimated that contribution from the other terms related to $J\left(\omega \right)$ at the higher frequencies is <1%.

### Expression of isotope-labelled MALT1(PCASP-Ig3)_339–719_ and preparation of NMR samples

The DNA sequence encoding for the PCASP and Ig3 domains of human MALT1, corresponding to residues 338–719 (Fig. 1d), and a C-terminal His6-tag was cloned into the expression vector pET21b (Novagen). The MALT1(PCASP-Ig3)_339–719_-his construct was transformed into *Escherichia coli* strain T7 express competent cells, and thereafter expressed in different isotopic labelling combinations in ^1/2^H, ^15^N, ^12/13^C-labelled M9 medium. Chemicals for isotope labelling (ammonium chloride, ^15^N (99%)), D-glucose, ^13^C (99%) and deuterium oxide were purchased from Cambridge Isotope Laboratories, Inc. The E549A mutation was introduced using the QuikChange Lightning kit (Agilent, Santa Clara, CA, USA). All cloned constructs and introduced modifications were verified by DNA sequencing. Both wild-type MALT1(PCASP-Ig3)_339–719_ and MALT1(PCASP-Ig3)_339–719_(E549A) were produced and purified as described here below. MALT1(PCASP-Ig3)_339–719_ was expressed in 1 L of D_2_O M9 medium using 3 g/L of U-[^13^C,^2^H]-glucose (CIL, Andover, MA) as the main carbon source, and 1 g/L of ^15^NH_4_Cl (CIL, Andover, MA) as the nitrogen source. One hour prior to induction, precursors were added to the growth medium as previously described^91^. For precursors, 70 mg/L alpha-ketobutyric acid, sodium salt (^13^C4, 98%, 3,3-^2^H, 98%) and 120 mg/L alpha-ketoisovaleric acid, sodium salt (1,2,3,4-^13^C4,99%, 3, 4, 4, 4, -^2^H 97%) (CIL, Andover, MA) were used. Bacterial growth was continued for 16 h at 16 °C and cells were thereafter harvested by centrifugation. Cells were resuspended in lysis buffer 20 mM TrisHCl (pH7.6), 150 mM NaCl, 2 mM DTT and lysed using an ultrasonicator, followed by centrifugation at 40,000 g for 30 min to remove cell debris. The supernatant was collected and incubated with Ni^2+^ Sepharose 6 Fast Flow (GE Healthcare) for 1 h at 4 °C. The target protein was eluted with a lysis buffer containing 200–500 mM imidazole. A Q-Sepharose HP column (GE Healthcare) was used to separate monomeric MALT1(PCASP-Ig3)_339–719_ from the dimer form. A final size exclusion chromatography (SEC) step was performed using a HiLoad 16/600 Superdex 200 prep grade column (GE Healthcare), with running buffer 20 mM HEPES 7.4, 50 mM NaCl, 1 mM DTT. The final monomer MALT1(PCASP-Ig3)_339–719_ protein sample was subsequently exchanged to a buffer (10 mM Tris 7.6, 50 mM NaCl, 2 mM TCEP, 0.002% NaN3, 10% D_2_O) suitable for NMR experiments using gravity flow PD10 desalting columns (GE Healthcare). Final yields from a four litres M9 culture were ~8 mg, and the purified MALT1(PCASP-Ig3)_339–719_-his tagged proteins were concentrated to at least 0.3–0.5 mM for NMR data acquisition.

A notable problem during data collection was the sample stability, manifested in a loss of signal intensity by ~ 20–30% over a period of 1 week. Nevertheless, the signal drop did not lead to systematic errors in the acquired data and analysis, but rather resulted in slightly more scattered and noisy data, which is expected when the signal-to-noise ratio diminishes. To overcome this problem, we performed the following steps: (i) all measurements were performed with a completely fresh MALT1(PCASP-Ig3)_339–719_ sample at each of the two fields, (ii) decrease of the signal intensity with time was not accompanied by any shift or broadening of the peaks, indicating that there was no reason to believe that the populations of protein conformations changed during the measurements, (iii) the measurements were performed using sorted NUS table, which converted the intensity drop into a small additional broadening of the ^15^N without any effect on the relaxation decay, (iv) the relaxation parameter vs residue over the two fields were consistent.

### NMR relaxation experiments and data processing

All NMR data were acquired at 298 K on 800 and 900 MHz Bruker spectrometers equipped with TCI cryo probes which are optimised for triple resonance experiments on biological macromolecules. All spectra were processed using either the mddnmr^92^ or the NMRPipe^93^ softwares at the NMRbox server (https://nmrbox.nmrhub.org/)^94^ or with the TopSpin 4.2.0 Bruker. To determine diffusion tensor MATLAB (MathWorks) and version 7 of the MATLAB-based ROTDIF^95,96^ were used. In house MATLAB scripts used for treatment of the ROTDIF-calculations and all subsequent data analyses are available upon request.

### Determination of ^15^N relaxation rates *R*_1_, *R*_2_ and ^15^N-(^1^H) nuclear Overhauser effect

A complete set of ^15^N relaxation rates *R*_1_, *R*_2_ and ^15^N-(^1^H) nuclear Overhauser effect (NOE) experiments for the backbone amides were acquired with the standard Bruker pulse sequences using TROSY (Transverse Relaxation-Optimised Spectroscopy) and sensitivity enhancement^97,98^ at 800 MHz and 900 MHz. The *R*_1_ and *R*_2_ spectra were recorded in the pseudo 3D mode with randomised order of the relaxation delays and the NUS table sorted in the ^15^N spectral dimension to mitigate signal decay during the experiment due to the slow sample degradation. The following 11 relaxation delays were used to measure *R*_1_ values at 800 MHz: 0.08, 0.16, 0.32, 0.48, 0.72, 0.88, 1.12, 1.36, 1.60, 2.00 and 2.40 s; and at 900 MHz: 0.08, 0.16, 0.32, 0.48, 0.72, 0.88, 1.12, 1.36, 1.76, 2.24 and 2.4 s. For the *R*_2_, the following nine relaxation delays were used at 800 MHz: 0.0058, 0.0115, 0.0173, 0.0230, 0.0288, 0.0346, 0.0403 s; and the following 11 relaxation delays were used at 900 MHz: 0.0058, 0.0115, 0.0173, 0.0230, 0.0288, 0.0346 and 0.0403 s, where each of the underlined delays were used twice. NOE experiments were recorded at 800 MHz and used the ^1^H saturation time of 5 s. Experimental parameters for *R*_1_, *R*_2_ and NOE measurements are summarised in Supplementary Table S5.

The relaxation rates were obtained by extracting peak volume from CcpNmr Analysis Assign^99^ while keeping the original peak position for all spectra in a relaxation decay data set. To extract the relaxation rates, mono-exponential functions were fitted to the relaxation decays using MATLAB’s built-in nonlinear regression programmes, with errors estimated from the covariance matrix. NOEs were calculated as the ratio of the peak intensities in the saturated and reference experiments, and the standard deviations for the NOEs were determined by propagating the errors of intensities estimated from the base plane noise determined from regions without any peaks. All error estimates are reported as one standard deviation (SD).

### Determination of ^15^N cross-correlated transverse relaxation of backbone

The ^15^N cross-correlated transverse relaxation experiment (CCR) was run for both MALT1(PCASP-Ig3)_339–719_ and mutated MALT1(PCASP-Ig3)_339–719_(E549A). The CCR experiment (Supplementary Table S5) was performed at 900 MHz using 2D ^1^H-^15^N TROSY and anti-TROSY experiments^100^ with relaxation delays Δ of 0 and 10 ms. The cross-correlation rates ${\eta }_{{xy}}$ were calculated from two peak intensities ${I}^{\alpha }$ in the TROSY spectrum, and ${I}^{\beta }$ in the anti-TROSY for Δ = 10 ms:

5 $${\eta }_{{xy}}=-{ln}({I}^{\beta }/{I}^{\alpha })/(2\varDelta )$$

The initial estimate of the error in ${\eta }_{{xy}}$ was propagated from the difference between *I*^*α/β*^ intensities measured for the relaxation delays Δ = 0. However, since the estimate from just two repeated measures was too crude, we averaged the errors obtained for MALT1(PCASP-Ig3)_339–719_ and MALT1(PCASP-Ig3)_339–719_(E549A). We also regularised the values by using a minimal relative error of 5%.

### Estimation of exchange contribution to the transverse relaxation rate *R*_2_

An assessment of the *R*_ex_-contribution to *R*_2_ can be made by comparing *R*_2_ with the transverse cross-correlated relaxation rate according to Eq. (1)^101^:

6 $${R}_{{ex}}={R}_{2}-[(3{d}^{2}+4{c}^{2})/\left(\right.2\sqrt{3{cd}}\left(3{cos }^{2}\theta -1\,\right)]{\eta }_{{xy}}$$

were, similarly to Eq. (4), we neglected the high frequency *J*(ω) terms that have very small contributions compared to the dominant *J*(0) and *J*(ω_*N*_) terms. The residue specific error in conformational exchange σ_Rex_ was determined by propagating the errors from *η*_xy_ and *R*_2_. In our qualitative analysis, we defined residues with statistically significant conformational exchange as those that show *R*_ex_/σ_Rex_ > 5.

### Calculation of Rotational diffusion tensor

ROTDIF takes the ^15^N relaxation parameters and the ^1^H-^15^N bond vector coordinates from the MALT1(PCASP-Ig3)_339–719_ crystal structure (PDB code 3V55) as input. Hydrogens were added to the crystal structure using the *addh* function in the Chimera 1.16 software^102^. For the illustration of the structures presented in this article, an AlphaFold-generated model was employed to replenish two regions (residues 495–497 and 469–480) within the PCASP domain that are missing in the crystal structure.

We used ROTDIF to fit the 900 MHz *R*_2_/*R*_1_, 800 MHz *R*_2_/*R*_1_ and 900 MHz CCR data to the axially symmetric model of MALT1(PCASP-Ig3)_339–719_, and obtained the principal values of two tensor components *D*_⊥_ and *D*_||_, and of the two Euler angles α and β. The standard errors of the fitted parameters were estimated using a resampling technique reminiscent of the *delete-d* jack-knife estimation^103^. Since the ROTDIF software requires *R*_2_/*R*_1_-ratios as input, we converted the ${\eta }_{{xy}}$-derived residue specific *τ*_C_, (Eq. 4) to residue-specific *R*_2_/*R*_1_-ratios for 900 MHz spectrometers by inverting Eq. (6) in^104^:

7 $$\frac{{R}_{2,i}}{{R}_{1,i}}=\frac{2}{3}{({\omega }_{N}{\tau }_{C,i})}^{2}+\frac{7}{6}$$

When calculating the residue specific *τ*_C_ using Eq. (4), we adjusted *S*^2^ – 0.88. This value was adjusted by equating the average *τ*_C_ obtained from the CCR-measurements (${\eta }_{{xy}}$) with those from the 800 and 900 MHz *R*_2_/*R*_1_-ratio measurements. The used *S*^2^ value is in line with the range 0.8–0.9 usually observed for structured parts of globular proteins and indicates a general good agreement between our ${\eta }_{{xy}}$ and *R*_2_/*R*_1_ data.

### Selection of residues for diffusion tensor calculations

The selection of residues for the ROTDIF-optimization in MATLAB constitutes a crucial step in our analysis. In this study, we applied the following four selection criteria for choosing the amide group of a specific amino acid residue for the calculation of the rotational diffusion of MALT1(PCASP-Ig3)339–719 : (i) we kept only residues with a relative *R*_2_/*R*_*1*_ error of below 10%, 15% and 25% in the 900 MHz, 800 MHz and CCR data sets, respectively; (ii) we excluded residues with B-factors for backbone nitrogen atoms in the crystal structure above 35 Å^2^. The average nitrogen backbone B-factor for the 368 residues (PDB code 3v55) is 33.3 ± 12 Å^2^, with a median of 29.6 Å^2^; (iii) we excluded residues whose peaks strongly overlapped in the ^1^H-^15^N TROSY spectrum; (iv) for reducing impact of milliseconds (conformational exchange) and nanosecond motions, we kept only residues within two standard deviations (SD) from the mean *R*_2_/*R*_1_-ratio for *R*_*2*_*, R*_*1*_ 800/900 MHz data and 1.7 SD from the mean *R*_2_/*R*_1_-ratio for the CCR-data. These four selection criteria reduced the number of residues from 270 assigned and characterised protein peaks to 116 (900 MHz *R*_2_/*R*_1_), 115 (800 MHz *R*_2_/*R*_1_) and 105 (900 MHz CCR). These three sets of selected peaks were used for the calculations of the rotational diffusion tensor.

### Estimation of diffusion tensors using statistical resampling

The experimental relaxation data for MALT1(PCASP-Ig3)_339–719_ have relatively poor signal-to-noise due to the large system size, and to degradation of the sample during experiment. The data quality worsens in the order *R*_2_/*R*_*1*_ 900 MHz → *R*_2_/*R*_*1*_ 800 MHz → CCR, and therefore we attributed the most explanatory power to the *R*_2_/*R*_*1*_ 900 MHz data. The two other sets of data, *R*_2_/*R*_*1*_ 800 MHz and CCR were used, extra carefully and mainly for the validation of the *R*_2_/*R*_*1*_ 900 MHz data. Furthermore, as far as we know, the method of calculation of the diffusion tensor from CCR data was not described before and thus requires validation on a well characterized protein system with high-quality experimental data. Thus, the rotation diffusion tensor results obtained from CCR are used in this study only qualitatively.

To perform the analysis with the noisy data we assessed the criteria for selecting amide HN pairs as outlined above, and performed the error analysis using the bootstrap resampling technique that resembles the *delete-d* jack-knife approach^105,106^. When the number of HN pairs used for the analysis exceeded 100 (*n* > 100), the rigorous delete-*d* jack-knife approach which exhaustively and systematically excludes all possible combinations of *d* samples from the set, requires too many parameter optimizations. A very similar result was obtained much faster by the statistical bootstrap sampling of a relatively small (*i.e*. 10^2^ in our case) number of parameter optimizations. The parameter uncertainties are approximated by $\sqrt{n/d}\,\times \sigma$, where in our case $\sigma$ is the standard deviation of a tensor component *D*_⊥_ or *D*_||_ obtained in 100 ROTDIF-simulations. The resampling method belongs to non-parametric statistics which do not require the assumption of normally distributed measurements. Statistical theory does not give a unified answer to what is a reasonable number for *d* and the degree of deletion seems to be model-dependent^107^. We found that omission *d* = 50% represented the lower limit, as no good fitting of the rotational diffusion tensor could be obtained below this level and we therefore used *d* = 20% for our calculations.

### Measurement of intramolecular interactions

Intramolecular amide-methyl interactions were confirmed by observing cross peaks in 3D SOFAST (SF), ^1^H-^15^N TROSY NOESY experiments. Additional intramolecular methyl-methyl interactions were obtained from 4D methyl-methyl SF HMQC-NOESY-HMQC experiments^108^. The acquisition parameters are presented in Supplementary Table S5.

### Chemical shift perturbation studies comparing wild-type MALT1(PCASP-Ig3)_339–719_ and mutated MALT1(PCASP-Ig3)_339–719_(E549A)

Assignment of backbone NH chemical shifts in the mutated MALT1(PCASP-Ig3)_339–719_(E549A) was based on assignment of the wild-type MALT1(PCASP-Ig3)_339–719_ previously reported by us^64^, and additional sets of 3D TA-acquisition experiments^109,110^, with parameters described in^64^ and deposited in the Biological Magnetic Resonance Data Bank (http://www.bmrb.wisc.edu/) with the BMRB accession code 52265. The new assignment was deposited into BMRB as an update of 52265 entry. Excellent reproducibility of the samples was confirmed by the perfect overlay of the spectra (Supplementary Fig. S1). Effective chemical shift perturbations were determined for the comparison of the chemical shifts of mutated MALT1(PCASP-Ig3)_339–719_(E549A) and wild type MALT1(PCASP-Ig3)_339–719_, and was calculated as Δδ_eff_ = ([Δδ(^1^H)]^2^ + [0.16Δδ(^15^N)]^2^)^1/2^.

### AlphaFold, PCA, ESMFold2, RoseTTAFold2 and NMA analyses

Ensembles of MALT1(PCASP-Ig3)_339–719_ structures were generated using AlphaFold version 2.3.1, and a massive sampling technique proposed by Wallner^60^. A total of 2000 structures were predicted with AF model *monomer_ptm* without templates, using dropouts at the inference stage. The dropout scheme was kept the same as during the AF training. The PCA^111^ was performed using the *Scikit-learn* Python library^112^. To analyse the set of structures, we performed the PCA in Cartesian coordinates for two sets of atoms: (a) PCAa, which includes the N, C_α_, C, O atoms of protein residues 344–717, excluding flexible N- and C-termini, and all side-chain heavy atoms of amino acids exhibiting multiple rotamers in χ_2_ torsion angles, including residue W580; (b) PCAb, akin to PCAa, but focusing only on the stretches of residues 579–584 and 652–659 including site chains of W580 and Y657. All structures were aligned using the PDB Superimpose module from Biopython (http://citebay.com/how-to-cite/biopython/). The alignment was done based on the C_α_ atoms of the stretches of residues 344–467, 484–500 and 510–562. These residues were selected for the performed alignment due to their location in the PCASP domain and high pLDDT scores.

For PCAa, the eigenvalues for the first three components were 0.48, 0.17, and 0.08. Thus, the analysis was limited to the 1^st^ and 2nd principal components. For PCAb, the eigenvalues for the first three components were 0.74, 0.13 and 0.04. We kept the first two principal components, which notably caught rotamer changes for residues W580 and Y657. An inverse transformation was applied to the first and second principal components, converting them back into their original 3D coordinates, and reconstructing the specific molecular motions they represent. Normal mode analysis (NMA) was performed using the online server elNémo^113,114^. The crystal structure of MALT1(PCASP-Ig3)_339–71_9 (PDB code 3V55) was used as input, and all programme parameters were left as default. The first three lowest frequency normal modes were used for further analysis. Both ESMFold2^79^ and RoseTTAFold2^80,81^ were used with default parameters to produce MALT1(PCASP-Ig3)_339–719_ structural models.

### Molecular dynamic *simulations*

MD simulations were performed in GROMACS version 2023.1^115^. All MD simulations were performed using the all-atom force field charmm36-mar2019_cufix.ff [https://github.com/intbio/gromacs_ff/tree/master/charmm36-mar2019_cufix.ff] with a refinement of the Lennard-Jones parameters (CUFIX)^116^ both for protein and, as recommended, TIP3P water model. For the initial structure, we used the MALT1(PCASP-Ig3)339–719 structure predicted by model 1.1 of AlphaFold^59^ using ColabFold version 1.5.5^117^ when run with default parameters without templates. The protein was centred in a periodic 104 Å cubic box, and 55 Na^+^ and 41 Cl^–^ ions were added to the system to emulate ionic strength and achieve electro-neutrality as the protein had a total charge of −14. Since the charge of the protein residues was calculated according to pH = 7.6, all histidine residues displayed neutral charges. Long-range electrostatic and van der Waals 10 Å cut-off were used. The structure was relaxed through a process called energy minimization to ensure a reasonable starting structure in terms of geometry and solvent orientation. Convergence was achieved at a maximum force of <1000 kJ/mol/nm in any atom. Equilibration was conducted in two phases, namely NVT ensemble (with protein molecular models heating from 0 – 300 K for 100 ps), and NPT ensemble for 1000 ns until the system became well equilibrated and reached a plateau in the root mean square deviation (RMSD) values (Supplementary Fig. S6). A Berendsen-type thermostat and barostat were used. Hydrogen-containing covalent bonds were constrained using the SHAKE algorithm and time steps of 2 fs were used. Following the equilibration, MD simulations were continued as a production run for 2000 ns under the same conditions. System stability was calculated using standard tools available in Gromacs^115^ including control of the temperature, pressure, energy, secondary structure, the box border, and RMSD. The PCA analysis was performed as described above for the AF structures.

### Reporting summary

Further information on research design is available in the Nature Portfolio Reporting Summary linked to this article.

### Supplementary information

Supplementary Information

Description of Additional Supplementary Files

Supplementary data

Reporting Summary

## Data Availability

The ensemble of AF and MD structures and other information and data are available upon request from the authors. The numerical sources for the graphs and plots are found in the supplementary data. The assignment is found in Biological Magnetic Resonance Data Bank (http://www.bmrb.wisc.edu/) with the BMRB accession code 52265. The data were acquired using TopSpin3.5 and TopSpin4.06 from Bruker.

## Code availability

The NMR spectra processing and analysis was performed with the following publicly available software NMRPipe 11.5, Mddnmr 2.7, TopSpin 4.2, CCPNMR 2.4.2, Bruker Dynamic Center 2.8, Matlab 7 (MathWorks), GROMACS 2023.1, AlphaFold 2.3.1, ColabFold 1.5.5, ESMFold2, RoseTTAFold2, ROTDIF 7, PremPS (https://lilab.jysw.suda.edu.cn/research/PremPS/). Information of the analysis protocols and relevant used parameters is found in the method section.

## References

[1] Hamp I, O’Neill TJ, Plettenburg O, Krappmann D (2021). A patent review of MALT1 inhibitors (2013-present). Expert Opin. Ther. Pat..

[2] Ruland J, Duncan GS, Wakeham A, Mak TW (2003). Differential requirement for Malt1 in T and B cell antigen receptor signaling. Immunity.

[3] Ruefli-Brasse AA, Lee WP, Hurst S, Dixit VM (2004). Rip2 participates in Bcl10 signaling and T-cell receptor-mediated NF-kappaB activation. J. Biol. Chem..

[4] Thome M (2008). Multifunctional roles for MALT1 in T-cell activation. Nat. Rev. Immunol..

[5] Hachmann J, Salvesen GS (2016). The paracaspase MALT1. Biochimie.

[6] Hailfinger S (2009). Essential role of MALT1 protease activity in activated B cell-like diffuse large B-cell lymphoma. Proc. Natl Acad. Sci. USA.

[7] Davis RE (2010). Chronic active B-cell-receptor signalling in diffuse large B-cell lymphoma. Nature.

[8] Rodriguez-Sevilla, J. J. & Salar, A. Recent advances in the genetic of MALT lymphomas. *Cancers* **14**, 176 (2022). ARTN 176. 10.3390/cancers14010176 PMC8750177 35008340

[9] Wang Y (2017). MALT1 promotes melanoma progression through JNK/c-Jun signaling. Oncogenesis.

[10] Ekambaram P (2018). The CARMA3-Bcl10-MALT1 signalosome drives NFkappaB activation and promotes aggressiveness in angiotensin II receptor-positive breast cancer. Cancer Res..

[11] Jacobs KA (2020). Paracaspase MALT1 regulates glioma cell survival by controlling endo-lysosome homeostasis. EMBO J.

[12] Solsona BG, Schmitt A, Schulze-Osthoff K, Hailfinger S (2022). The paracaspase MALT1 in cancer. Biomedicines.

[13] O’Neill TJ, Tofaute MJ, Krappmann D (2023). Function and targeting of MALT1 paracaspase in cancer. Cancer Treat. Rev..

[14] Jaworski M (2014). Malt1 protease inactivation efficiently dampens immune responses but causes spontaneous autoimmunity. EMBO J..

[15] Gewies A (2014). Uncoupling Malt1 threshold function from paracaspase activity results in destructive autoimmune inflammation. Cell Rep..

[16] Howes A (2016). Psoriasis mutations disrupt CARD14 autoinhibition promoting BCL10-MALT1-dependent NF-kappaB activation. Biochem. J..

[17] Afonina IS (2016). The paracaspase MALT1 mediates CARD14-induced signaling in keratinocytes. Embo Rep..

[18] O’Neill TJ, Gewies A, Seeholzer T, Krappmann D (2023). TRAF6 controls T cell homeostasis by maintaining the equilibrium of MALT1 scaffolding and protease functions. Front. Immunol..

[19] Ruland J, Hartjes L (2019). CARD-BCL-10-MALT1 signalling in protective and pathological immunity. Nat. Rev. Immunol..

[20] Qiao Q (2013). Structural architecture of the CARMA1/Bcl10/MALT1 signalosome: nucleation-induced filamentous assembly. Mol Cell.

[21] Li D, Wang YL (2018). Coordination of cell migration mediated by site-dependent cell-cell contact. Proc. Natl Acad. Sci. USA.

[22] Jaworski M, Thome M (2016). The paracaspase MALT1: biological function and potential for therapeutic inhibition. Cell Mol. Life Sci..

[23] Rebeaud F (2008). The proteolytic activity of the paracaspase MALT1 is key in T cell activation. Nat. Immunol..

[24] Coornaert B (2008). T cell antigen receptor stimulation induces MALT1 paracaspase-mediated cleavage of the NF-kappa B inhibitor A20. Nat. Immunol..

[25] Schlauderer F (2018). Molecular architecture and regulation of BCL10-MALT1 filaments. Nat. Commun..

[26] Uren AG (2000). Identification of paracaspases and metacaspases: two ancient families of caspase-like proteins, one of which plays a key role in MALT lymphoma. Mol. Cell.

[27] Wiesmann C (2012). Structural determinants of MALT1 protease activity. J. Mol. Biol..

[28] Yu JW, Jeffrey PD, Ha JY, Yang XL, Shi YG (2011). Crystal structure of the mucosa-associated lymphoid tissue lymphoma translocation 1 (MALT1) paracaspase region. Proc. Natl Acad. Sci. USA.

[29] Hachmann J (2012). Mechanism and specificity of the human paracaspase MALT1. Biochem. J..

[30] Pelzer C (2013). The protease activity of the paracaspase MALT1 is controlled by monoubiquitination. Nat. Immunol..

[31] Roschitzki-Voser, H. et al. Human caspases: expression, purification and kinetic characterization. *Protein Expr. Purif.* **84**, 236–246 (2012). 10.1016/j.pep.2012.05.009 22683476

[32] Boatright KM (2003). A unified model for apical caspase activation. Mol. Cell.

[33] Cabalzar K (2013). Monoubiquitination and activity of the paracaspase MALT1 requires glutamate 549 in the dimerization interface. PLoS One.

[34] Snipas SJ (2004). Characteristics of the caspase-like catalytic domain of human paracaspase. Biol. Chem..

[35] Schairer R (2020). Allosteric activation of MALT1 by its ubiquitin-binding Ig3 domain. Proc. Natl Acad. Sci. USA.

[36] Schlauderer F (2013). Structural analysis of phenothiazine derivatives as allosteric nhibitors of the MALT1 paracaspase. Angew. Chem. Int. Edit.

[37] Quancard J (2019). An allosteric MALT1 inhibitor is a molecular corrector rescuing function in an immunodeficient patient. Nat. Chem. Biol..

[38] Changeux JP, Edelstein SJ (2005). Allosteric mechanisms of signal transduction. Science.

[39] Motlagh HN, Wrabl JO, Li J, Hilser VJ (2014). The ensemble nature of allostery. Nature.

[40] Jiang Y, Kalodimos CG (2017). NMR studies of large proteins. J. Mol. Biol..

[41] Wand AJ (2001). Dynamic activation of protein function: a view emerging from NMR spectroscopy. Nat. Struct. Biol..

[42] Tsai CJ, del Sol A, Nussinov R (2008). Allostery: Absence of a change in shape does not imply that allostery is not at play. J. Mol. Biol..

[43] Taly A (2006). Implications of the quaternary twist allosteric model for the physiology and pathology of nicotinic acetylcholine receptors. Proc. Natl Acad. Sci. USA.

[44] Wodak SJ (2019). Allostery in its many disguises: from theory to applications. Structure.

[45] Zhuravleva A (2007). Propagation of dynamic changes in barnase upon binding of barstar: an NMR and computational study. J. Mol. Biol..

[46] Henzler-Wildman K, Kern D (2007). Dynamic personalities of proteins. Nature.

[47] Wieteska L, Shahidi S, Zhuravleva A (2017). Allosteric fine-tuning of the conformational equilibrium poises the chaperone BiP for post-translational regulation. Elife.

[48] Strotz D (2020). Protein allostery at atomic resolution. Angew. Chem. Int. Edit..

[49] Köhler C (2020). Dynamic allosteric communication pathway directing differential activation of the glucocorticoid receptor. Sci. Adv..

[50] Toyama Y, Kay LE (2021). Probing allosteric interactions in homo-oligomeric molecular machines using solution NMR spectroscopy. Proc. Natl Acad. Sci. USA.

[51] Astore MA, Pradhan AS, Thiede EH, Hanson SM (2024). Protein dynamics underlying allosteric regulation. Curr. Opin. Struct. Biol..

[52] Palmer AG (2004). NMR characterization of the dynamics of biomacromolecules. Chem. Rev..

[53] Mittermaier AK, Kay LE (2009). Observing biological dynamics at atomic resolution using NMR. Trends Biochem. Sci..

[54] Baldwin AJ, Kay LE (2009). NMR spectroscopy brings invisible protein states into focus. Nat. Chem. Biol..

[55] Wand AJ (2013). The dark energy of proteins comes to light: conformational entropy and its role in protein function revealed by NMR relaxation. Curr. Opin. Struc. Biol..

[56] Tzeng SR, Kalodimos CG (2011). Protein dynamics and allostery: an NMR view. Curr. Opin. Struct. Biol..

[57] Shukla VK, Siemons L, Hansen DF (2023). Intrinsic structural dynamics dictate enzymatic activity and inhibition. Proc. Natl Acad. Sci..

[58] Motlagh HN, Li J, Thompson EB, Hilser VJ (2012). Interplay between allostery and intrinsic disorder in an ensemble. Biochem. Soc. T..

[59] Jumper J (2021). Highly accurate protein structure prediction with AlphaFold. Nature.

[60] Wallner, B. Improved multimer prediction using massive sampling with AlphaFold in CASP15. *Proteins*10.1002/prot.26562 (2023). 10.1002/prot.26562 37548092

[61] Ma PY, Li DW, Bruschweiler R (2023). Predicting protein flexibility with AlphaFold. Proteins Struct. Funct. Bioinform..

[62] del Alamo D (2022). Integrated AlphaFold2 and DEER investigation of the conformational dynamics of a pH-dependent APC antiporter. Proc. Natl Acad. Sci. USA.

[63] Wayment-Steele, H. K. et al. Predicting multiple conformations via sequence clustering and AlphaFold2. *Nature* **625**, 832–839 (2023). 10.1038/s41586-023-06832-9 PMC10808063 37956700

[64] Unnerstale, S. et al. Backbone assignment of the MALT1 Paracaspase by Solution NMR. *PLoS One*10.1371/journal.pone.0146496 (2016). 10.1371/journal.pone.0146496 PMC4720288 26788853

[65] Han, X. et al. Assignment of IVL-Methyl side chain of the ligand-free monomeric human MALT1 paracaspase-IgL(3) domain in solution. *Biomol. NMR. Assign.* **16**, 363–371 (2022). 10.1007/s12104-022-10105-3 PMC9510110 36094731

[66] Qiu L, Dhe-Paganon S (2011). Oligomeric structure of the MALT1 tandem Ig-like domains. PLoS One.

[67] Cavanagh, J., Fairbrother, W., Palmer III, A., Rance, M. & Skelton, N. *Protein NMR Spectroscopy* (Elsevier Academic Press, 2007).

[68] Barbato G, Ikura M, Kay LE, Pastor RW, Bax A (1992). Backbone dynamics of calmodulin studied by N-15 relaxation using inverse detected 2-dimensional Nmr-spectroscopy - the central helix is flexible. Biochem. US.

[69] Brüschweiler R, Liao X, Wright PE (1995). Long-range motional restrictions in a multidomain zinc-finger protein from anisotropic tumbling. Science.

[70] Fushman D, Varadan R, Assfalg M, Walker O (2004). Determining domain orientation in macromolecules by using spin-relaxation and residual dipolar coupling measurements. Prog. Nucl. Mag. Res. Sp.

[71] Orekhov VY, Nolde D, Golovanov A, Korzhnev D, Arseniev A (1995). Processing of heteronuclear NMR relaxation data with the new software DASHA. Appl. Mag. Reson..

[72] Korzhnev DM, Billeter M, Arseniev AS, Orekhov VY (2001). NMR studies of Brownian tumbling and internal motions in proteins. Prog. Nucl. Magn. Reson. Spectrosc..

[73] Orekhov, V. Y., Korzhnev, D. M., Pervushin, K. V., Hoffmann, E. & Arseniev, A. S. Sampling of protein dynamics in nanosecond time scale by N NMR relaxation and self-diffusion measurements. *J. Biomol. Struct. Dyn.* **17**, 157–174 (1999). 10.1080/07391102.1999.10508348 10496429

[74] Mariani V, Biasini M, Barbato A, Schwede T (2013). lDDT: a local superposition-free score for comparing protein structures and models using distance difference tests. Bioinformatics.

[75] Chen Y (2020). PremPS: Predicting the impact of missense mutations on protein stability. PLoS Comput. Biol..

[76] Sefer AP (2022). Expanding the clinical and immunological phenotypes and natural history of MALT1 deficiency. J. Clin. Immunol..

[77] Zinovjev K (2024). Activation and friction in enzymatic loop opening and closing dynamics. Nat. Commun..

[78] Yonetani T, Laberge M (2008). Protein dynamics explain the allosteric behaviors of hemoglobin. Bba Proteins Proteom.

[79] Lin ZM (2023). Evolutionary-scale prediction of atomic-level protein structure with a language model. Science.

[80] Baek, M. et al. Efficient and accurate prediction of protein structure using RoseTTAFold2. *bioRxiv*10.1101/2023.05.24.542179 (2023).

[81] Liu S, Wu K, Chen C (2022). Obtaining protein foldability information from computational models of AlphaFold2 and RoseTTAFold. Comput. Struct. Biotec..

[82] Gunther, H. *NMR Spectroscopy. An Introduction*. https://vikramuniv.ac.in/files/wp-content/uploads/M._Sc._II_SEM-Paper_IV-Unit_I-NMR-Part_I-Dr_Darshana_Mehta.pdf (1987).

[83] Unnerstale, S. et al. In *The XXVIIth International Conference on Magnetic Resonance in Biological Systems* (Kyoto International Conference Center, 2016).

[84] Nussinov, R., Zhang, M. Z., Liu, Y. L. & Jang, H. AlphaFold, allosteric, and orthosteric drug discovery: Ways forward. *Drug Discov. Today* **28**, 103551 (2023). 10.1016/j.drudis.2023.103551 PMC10238671 36907321

[85] Carugo O (2023). pLDDT values in AlphaFold2 protein models are unrelated to globular protein local flexibility. Crystals.

[86] Terwilliger, T. C. et al. AlphaFold predictions are valuable hypotheses and accelerate but do not replace experimental structure determination. *Nat. Methods* **21**, 110–116 (2023). 10.1038/s41592-023-02087-4 PMC10776388 38036854

[87] Jarymowycz VA, Stone MJ (2006). Fast time scale dynamics of protein backbones: NMR relaxation methods, applications, and functional consequences. Chem. Rev..

[88] Luginbühl, P. & Wüthrich, K. Semi-classical nuclear spin relaxation theory revisited for use with biological macromolecules. *Prog. Nucl. Mag. Res. Sp* **40**, 199-247 (2002).

[89] Lee D, Hilty C, Wider G, Wüthrich K (2006). Effective rotational correlation times of proteins from NMR relaxation interference. J. Magn. Reson..

[90] Robson SA, Dağ Ç, Wu H, Ziarek JJ (2021). TRACT revisited: an algebraic solution for determining overall rotational correlation times from cross-correlated relaxation rates. J. Biomol. NMR.

[91] Tugarinov V, Kanelis V, Kay LE (2006). Isotope labeling strategies for the study of high-molecular-weight proteins by solution NMR spectroscopy. Nat. Protoc..

[92] Orekhov V, Jaravine VA (2011). Analysis of non-uniformly sampled spectra with multi-dimensional decomposition. Prog. Nucl. Mag. Res. Sp.

[93] Delaglio F (1995). Nmrpipe - a multidimensional spectral processing system based on UNIX pipes. J. Biomol. NMR.

[94] Maciejewski MW (2017). NMRbox: A resource for bomolecular NMR computation. Biophys. J..

[95] Walker O, Varadan R, Fushman D (2004). Efficient and accurate determination of the overall rotational diffusion tensor of a molecule from <SUP > 15 < /SUP > N relaxation data using computer program ROTDIF. J. Magn. Reson..

[96] Fushman D (2002). Determination of protein dynamics using 15 N relaxation measurements. BioNMR Drug Res..

[97] Lakomek NA, Ying JF, Bax A (2012). Measurement of N relaxation rates in perdeuterated proteins by TROSY-based methods. J. Biomol. NMR.

[98] Zhu G, Xia Y, Nicholson LK, Sze KH (2000). Protein dynamics measurements by TROSY-based NMR experiments. J. Magn. Reson..

[99] Skinner SP (2016). CcpNmr analysis assign: a flexible platform for integrated NMR analysis. J. Biomol. NMR.

[100] Vallurupalli P, Hansen DF, Stollar E, Meirovitch E, Kay LE (2007). Measurement of bond vector orientations in invisible excited states of proteins. Proc. Natl Acad. Sci. USA.

[101] Palmer AG, Kroenke CD, Loria JP (2001). Nuclear magnetic resonance methods for quantifying microsecond-to-millisecond motions in biological macromolecules. Method Enzymol..

[102] Pettersen EF (2004). UCSF chimera - a visualization system for exploratory research and analysis. J Comput Chem.

[103] Mosteller, F. & Tukey, J. W. *Data Analysis and Regression: A Second Course in Statistics* 1st edn, Vol. 608 (Pearson, 1977).

[104] Fushman D, Weisemann R, Thuring H, Ruterjans H (1994). Backbone dynamics of ribonuclease-T1 and its complex with 2’gmp studied by 2-dimensional heteronuclear Nmr-spectroscopy. J. Biomol. NMR.

[105] Bonamente, M. *Statistics and Analysis of Scientific Data* 1st edn, XV, Vol. 301 (Springer, 2017).

[106] Zaman T, Alakus K (2015). Analysis of the invariance and generalizability of multiple linear regression model results obtained from Maslach burnout scale through jackknife method. Open J. Stat..

[107] Shi XQ (1988). A note on the delete-D jackknife variance estimators. Stat. Probabil. Lett..

[108] Zwahlen C (1998). An NMR experiment for measuring methyl-methyl NOEs in C-13-labeled proteins with high resolution. J. Am. Chem. Soc..

[109] Isaksson L (2013). Highly efficient NMR assignment of intrinsically disordered proteins: application to B- and T cell receptor domains. PLoS One.

[110] Jaravine V, Zhuravleva A, Permi P, Ibraghimov I, Orekhov VY (2008). Hyper-dimensional NMR spectroscopy with nonlinear sampling. J. Am. Chem. Soc..

[111] Kitao A, Go N (1999). Investigating protein dynamics in collective coordinate space. Curr. Opin. Struc. Biol..

[112] Pedregosa F (2011). Scikit-learn: machine learning in Python. J. Mach. Learn. Res..

[113] Suhre K, Sanejouand YH (2004). ElNemo: a normal mode web server for protein movement analysis and the generation of templates for molecular replacement. Nucleic Acids Res..

[114] Bauer JA, Pavlovic J, Bauerová-Hlinková V (2019). Normal mode analysis as a routine part of a structural investigation. Molecules.

[115] Abraham, M. J. et al. GROMACS: High performance molecular simulations through multi-level parallelism from laptops to supercomputers. *SoftwareX* **1**-**2**, 19–25 (2015).

[116] Yoo J, Aksimentiev A (2018). New tricks for old dogs: improving the accuracy of biomolecular force fields by pair-specific corrections to non-bonded interactions. Phys. Chem. Chem. Phys..

[117] Mirdita M (2022). ColabFold: making protein folding accessible to all. Nat. Methods.

