# Supplementary material for: Insights into mechanisms of MALT1 allostery from NMR and AlphaFold dynamic analyses
Source: Commun Biol. 2024 Jul 16;7:868. doi: 10.1038/s42003-024-06558-y (PMC11252132; doi:10.1038/s42003-024-06558-y)
Supplement: Supplementary file 1 — Supplementary Information [file 42003_2024_6558_MOESM1_ESM.pdf]

Supporting Information

Insights into mechanisms of MALT1 allostery from NMR and AlphaFold dynamic analyses

Johan Wallerstein[1], Xiao Han[2,3], Maria Levkovets[4], Dmitry Lesovoy[5], Daniel Malmodin[4], Claudio Mirabello[6,7], Björn Wallner[7], Renhua Sun[2,3], Tatyana Sandalova[2,3], Peter Agback[8], Göran Karlsson[1,4], Adnane Achour[2,3], Tatiana Agback[8, †*], Vladislav Orekhov[1,4, ,†*]

Author affiliations:

[1]Department of Chemistry and Molecular Biology, University of Gothenburg, Box 465, SE-40530 Gothenburg, Sweden.

[2]Science for Life Laboratory, Department of Medicine, Solna, Karolinska Institute SE-17165 Solna

[3] Division of Infectious Diseases, Karolinska University Hospital, SE-171 76 Stockholm, Sweden.

[4]Swedish NMR Centre, University of Gothenburg, Box 465, SE-40530 Gothenburg, Sweden.

[5]Shemyakin-Ovchinnikov Institute of Bioorganic Chemistry RAS, 117997 Moscow, Russia.

[6] Dept of Physics, Chemistry and Biology, Linköping University, 581 83 Linköping, Sweden

[7] National Bioinformatics Infrastructure Sweden, Science for Life Laboratory, Sweden

[8]Department of Molecular Sciences, Swedish University of Agricultural Sciences, PO Box 7015, SE-750 07 Uppsala, Sweden.

[†]These authors jointly supervised this work

* Corresponding authors: tatiana.agback@slu.se, vladislav.orekhov@nmr.gu.se

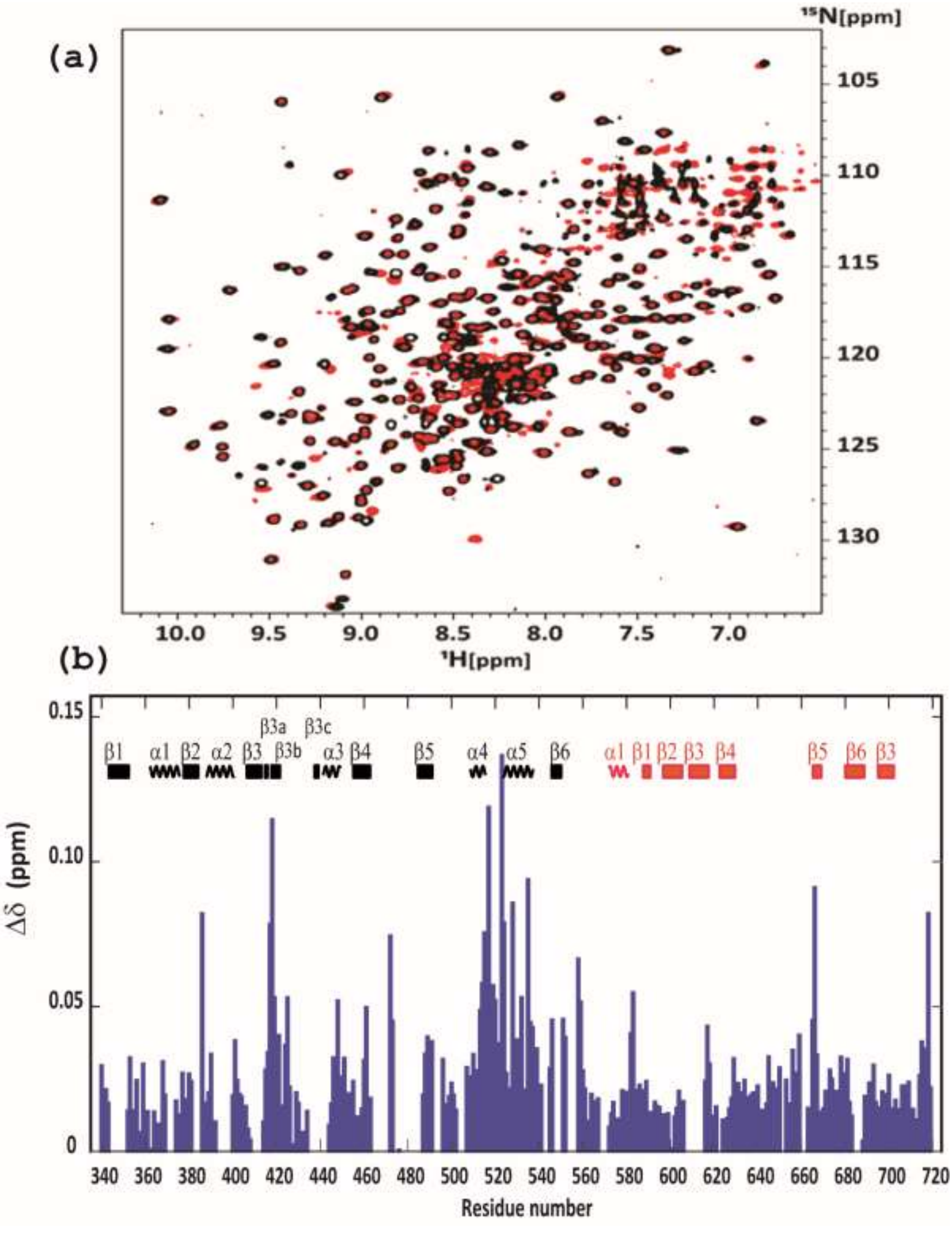

Figure S1. Chemical shift difference for the wild time and E549A mutant of MALT1(PCASP-Ig3)$_{339–719}$ (a) The superposition of the TROSY ^{1}H-^{15}N spectra of the apo form of MALT1(PCASP-Ig3)$_{339–719}$ (black) and its mutated variant E549A (red) recorded at 25 °C on a 900 MHz spectrometer. **(b)** Chemical shift perturbations for wild-type MALT1(PCASP-Ig3)$_{339–719}$ (blue circle) and MALT1(PCASP-Ig3)$_{339–719}$(E549A). Secondary structure elements are indicated and numbered for each domain at the top with PCASP in black and Ig3 in red.

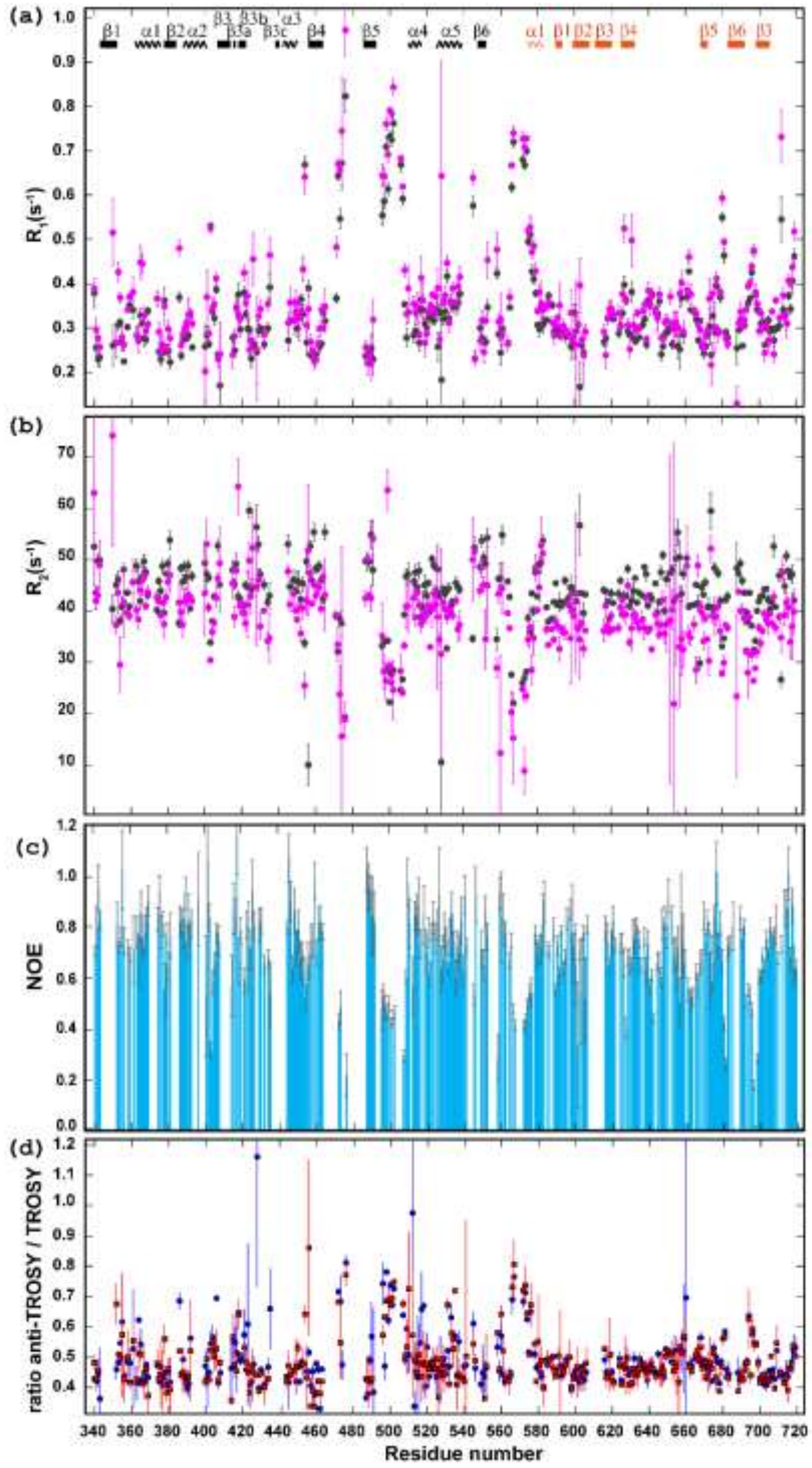

Figure S2. (a) ^{15}N R_1-relaxation rates and **(b)** ^{15}N R_2-relaxation rates for MALT1(PCASP-Ig3)$_{339–719}$. Black dots represent 900 MHz data and magenta dots represent 800 MHz data. Secondary structure elements are indicated and numbered for each domain at the top. The start of the α1 helix at residue 574 marks the separation between the PCASP and Ig3 domains. Error bars show 1 SD. **(c)** ^{15}N-(^{1}H) NOE with error bars from 800 MHz. **(d)** ^{15}N anti-TROSY/TROSY ratio for MALT1(PCASP-Ig3)$_{339–719}$ (blue circle) and for MALT1(PCASP-Ig3)$_{339–719}$(E549A) (red square). Data sets are from 900 MHz. Error bars show 1 SD calculated from the two intensities at $t_{CPMG} = 0$ for the anti-TROSY and the TROSY data sets. Secondary structure elements are indicated and numbered for each domain at the top with PCASP in black and Ig3 in red.

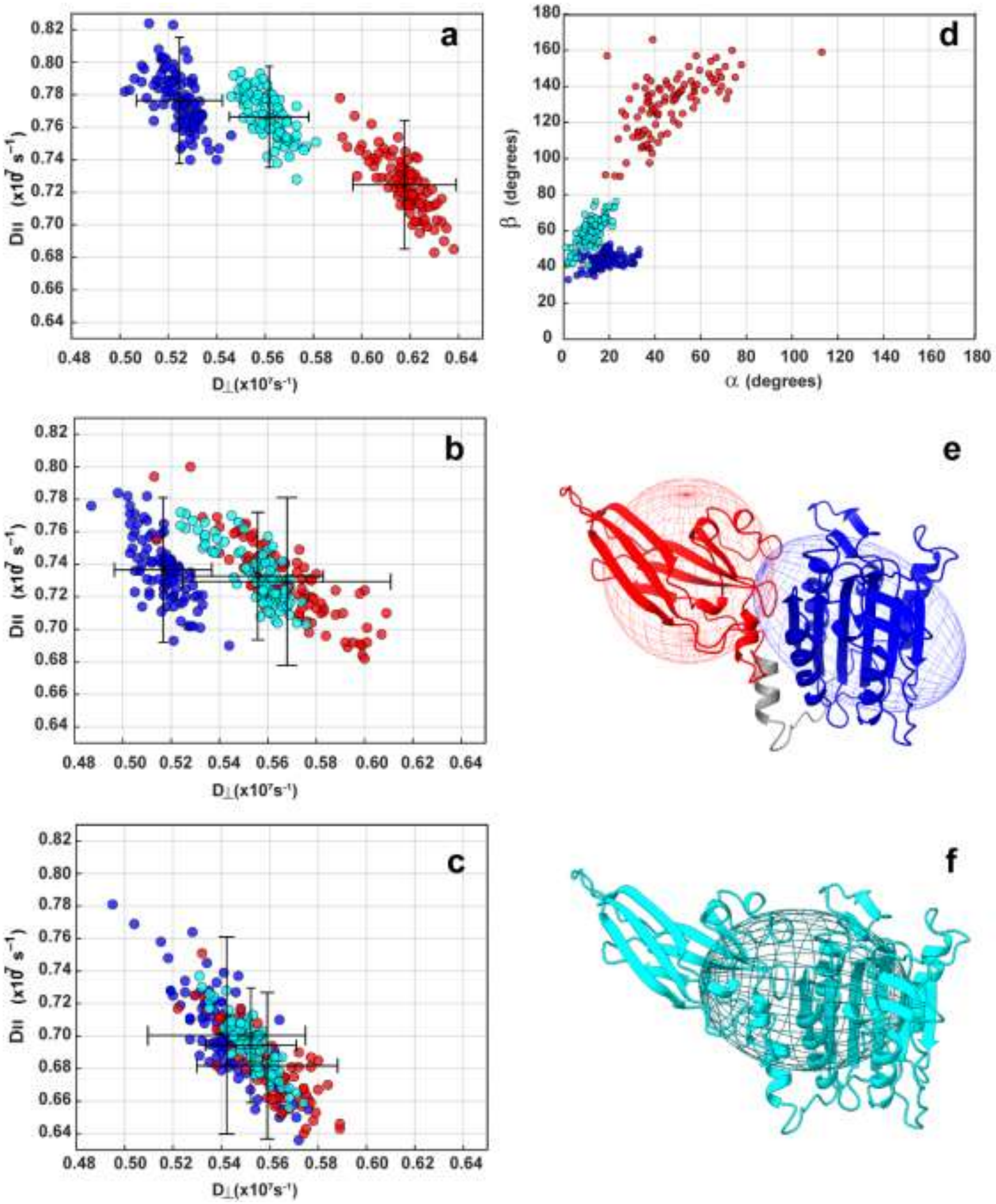

Figure S3. Diffusion tensor components D_{ZZ} and $D_{XX}D_{YY}$ from resampled ROTDIF optimisations.

Each red dot corresponds to a tensor calculated for one resampling instance for the Ig3 domain, the blue dots represent the PCASP domain and cyan dots represent the full MALT1(PCASP-Ig3)$_{339–719}$ for **(a)** 900 MHz ^{15}N R_2/R_1 data, **(b)** 800 MHz ^{15}N R_2/R_1 data, **(c)** 900 MHz cross-correlated transverse relaxation data (CCR). Error bars in (a)–(c) show one SD and are calculated taking the resampling fraction of d=20% into account. **(d)** α- and β-angles for the diffusion tensors for the 900 MHz ^{15}N R_2/R_1 data set. **(e)** Ellipsoid representation of the diffusion tensors (900 MHz ^{15}N R_2/R_1 data) mapped on the crystal structure of MALT1(PCASP-Ig3)$_{339–719}$, with Ig3 depicted in red and PCASP in blue. The grey part, residue 563–582, comprising the loop and the α1 helix is excluded from the optimization in ROTDIF. See material and method section for the procedure of selection residues in the respective domains. The principal axis of the axially symmetric diffusion tensors crosses the poles of the ellipsoids. **(f)** The diffusion tensor calculated for the full protein.

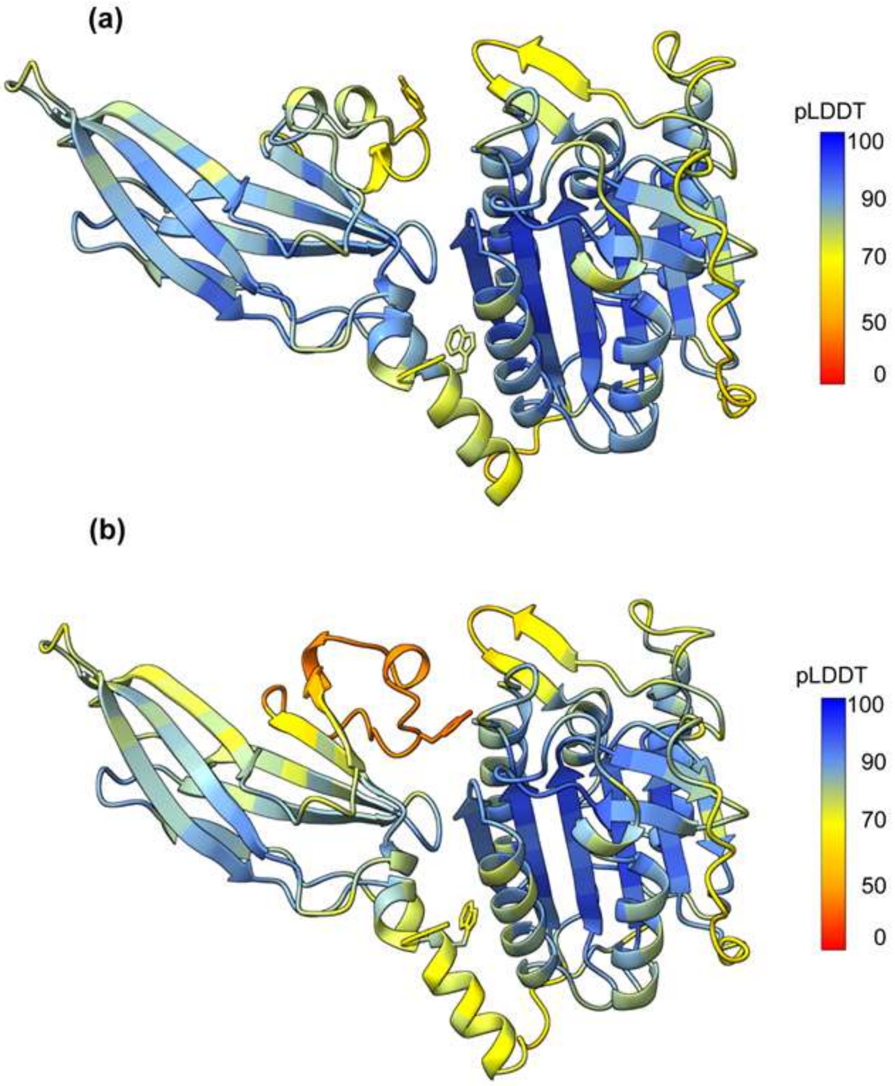

Figure S4. Molecular models of MALT1(PCASP-Ig3)$_{339–719}$ predicted by **(a)** RoseTTAFold2 and **(b)** ESMFold2. Per-residue RoseTTAFold2 and ESMFold2 confidence scores (pLDDT) are mapped on the structures. The low pLDDT scores of 0-50 indicate conformation heterogeneity.

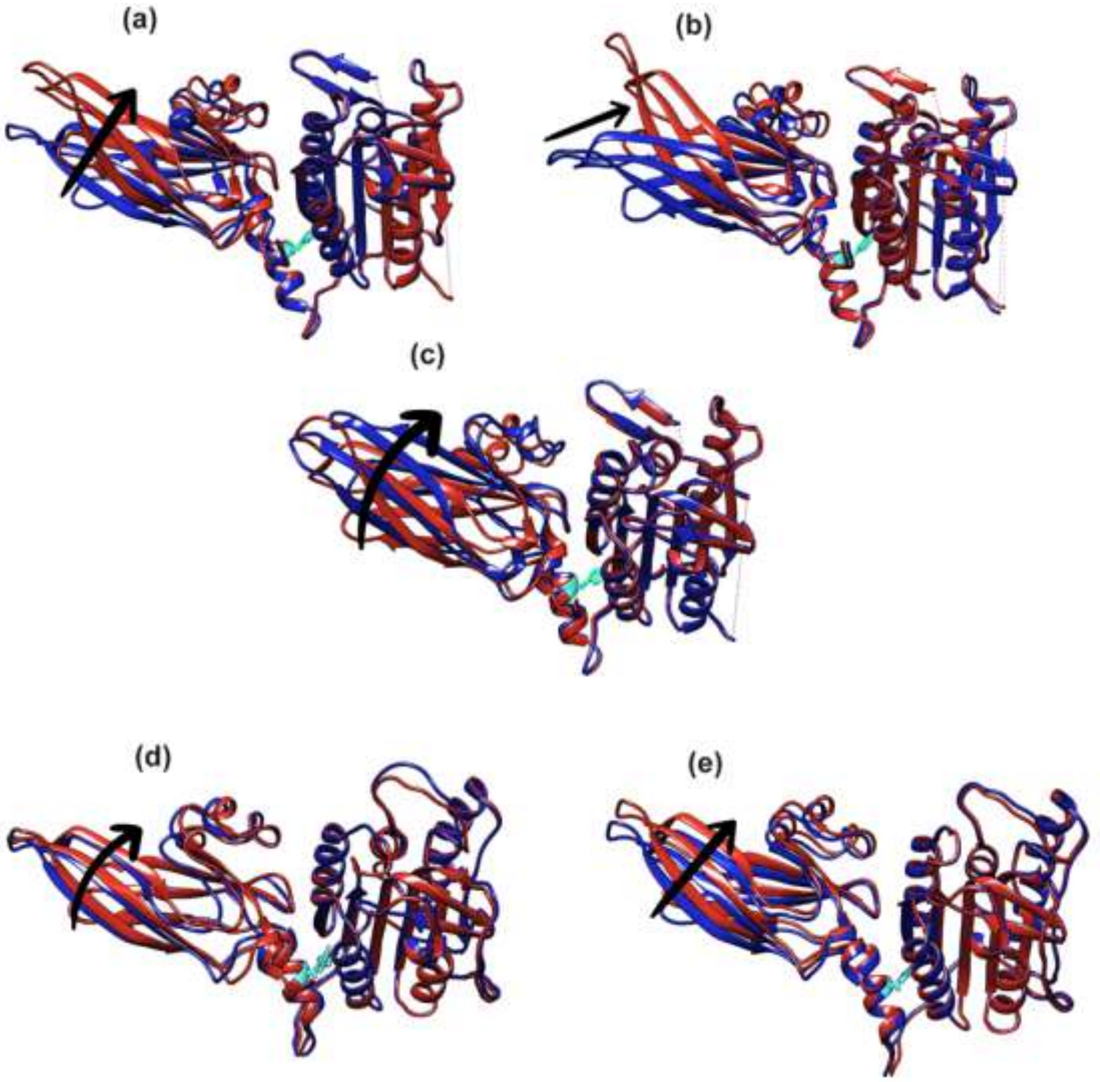

Figure S5. (a), **(b)**, **(c)** Molecular models of MALT1(PCASP-Ig3)$_{339–719}$ predicted using the NMA showing modes 7, 8 and 9, respectively. **(d)**, **(e)** PC1 and PC2 modes, respectively, obtained on confirmation ensemble of 2000 structures extracted from MD trajectory of 2 μs after 1 μs equilibration**.**

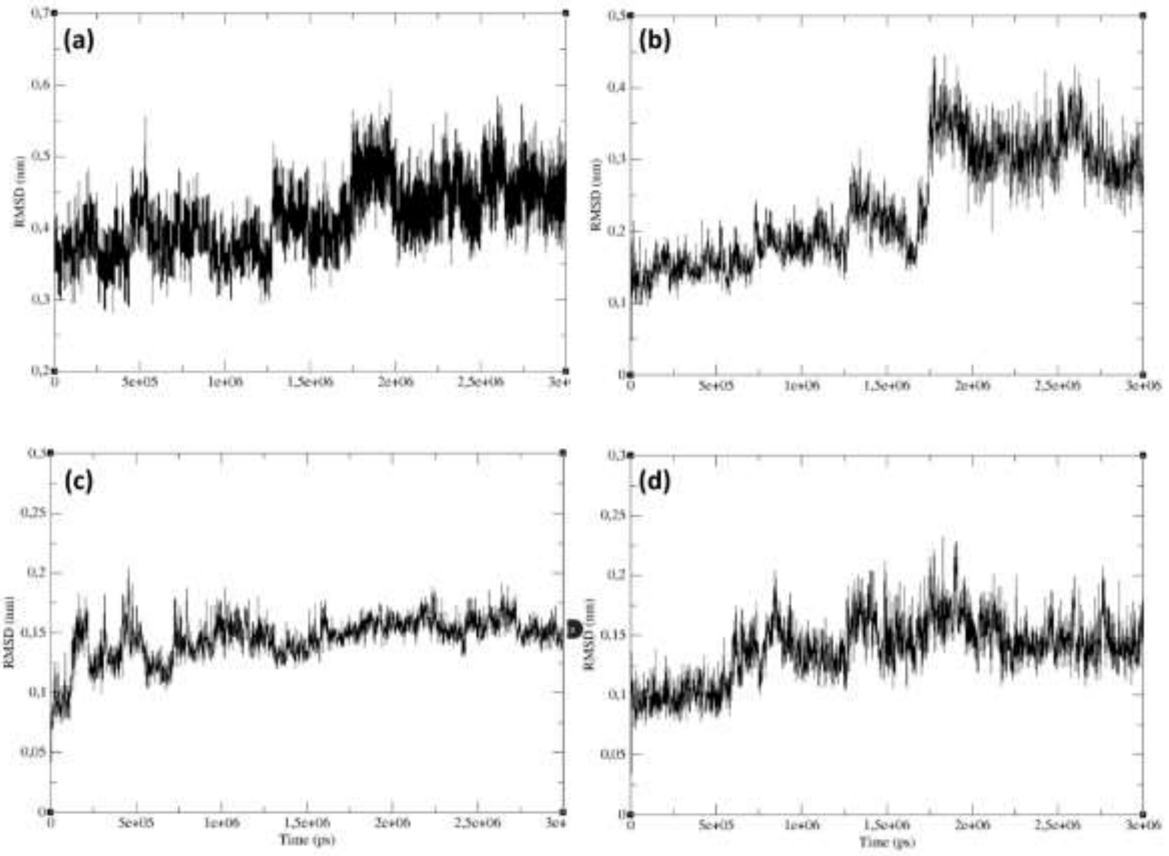

Figure S6. RMSD along the MD trajectory.

RMSD vs the initial structure for the backbone heavy atoms of MALT1(PCASP-Ig3)$_{339–719}$ obtained during 3us MD trajectory. 2 us production run used for the analysis started after the first 1us equilibration. The starting structure was obtained from AF of MALT1(PCASP-Ig3)$_{339–719}$. **a**) RMSD over trajectory of MALT1(PCASP-Ig3)$_{339–719}$ calculated using all amino acids, **b**) the RMSD calculated with exclusion of mobile N- and C-termini 1-4 and 381-388, the mobile linker between domains 226-234, and mobile loops 131-146 and 155-171. Note a significant decrease in RMSD compared to panel **a**, indicating a substantial contribution of the disordered region movements to RMSD values in panel **a**. Additionally, the exclusion of mobile regions led to a more noticeable relative movement of domains in panel b. Panels **c**) and **d**) depict RMSD separately for the first and second domains, respectively, excluding mobile regions, indicating the relative stability of the hydrophobic core of both domains.

Table S1. ^{15}N backbone relaxation rates R_1, σ_{R1}, R_2, σ_{R2} for 900 MHz and 800 MHz. 'ROTDIF' columns mark by 1 (0) residues used (not used) in the rotational diffusion tensor calculation.

Residue	Type	900 MHz					800 MHz				
		R_1	σ_{R1}	R_2	σ_{R2}	ROTDIF	R_1	σ_{R1}	R_2	σ_{R2}	ROTDIF
340	A	0.38	0.03	53	3	0	0.39	0.02	63	20	0
341	K	0.26	0.03	44	1	0	0.30	0.03	42	2	1
342	D	0.23	0.02	50	1	1	0.28	0.02	43	2	1
343	K	0.23	0.02	50	1	1	0.26	0.02	49	5	1
350	N	0.26	0.05	40	3	0	0.52	0.08	74	20	0
352	N	0.27	0.03	45	2	0	0.27	0.04	37	2	1
353	Y	0.31	0.01	46	1	0	0.43	0.02	44	4	1
354	R	0.31	0.01	38	1	0	0.37	0.01	30	6	0
355	E	0.27	0.02	41	2	0	0.28	0.02	-	-	0
356	H	0.22	0.01	48	1	0	0.26	0.02	42	7	0
358	K	0.30	0.01	43.5	0.4	0	0.34	0.01	40	1	0
359	L	0.35	0.01	40.8	0.4	1	0.37	0.01	39	1	1
361	A	0.34	0.02	46	2	1	0.38	0.03	46	5	0
363	L	0.28	0.01	49	1	1	0.28	0.02	40	1	0
364	V	0.27	0.03	46	2	0	0.28	0.03	38	2	0
365	D	0.33	0.02	42	2	1	0.45	0.04	44	2	0
366	V	0.31	0.01	47	1	1	0.45	0.02	46	3	1
367	Y	0.28	0.02	50	1	1	0.33	0.02	47	1	1
368	E	0.27	0.01	47	1	1	0.29	0.02	41	1	1
369	L	0.34	0.01	43	1	1	0.31	0.01	44	1	0
374	R	0.37	0.01	40	1	1	0.37	0.02	43	1	1
375	Q	0.25	0.01	46	1	1	0.29	0.02	42	1	1
376	L	0.23	0.01	44	2	0	0.25	0.03	39	1	1
377	D	0.24	0.01	49	1	1	0.26	0.02	41.6	0.4	1
378	F	0.36	0.03	40	2	1	0.35	0.04	37	3	1
379	K	0.25	0.01	47	1	0	0.25	0.01	43	1	0
380	V	0.25	0.02	49	1	1	0.30	0.02	46	1	1
381	V	0.22	0.02	54	2	1	0.28	0.02	47	3	1
386	L	0.37	0.01	38	1	0	0.48	0.02	42	4	0
387	T	0.24	0.01	48	1	1	0.26	0.02	40	2	0
388	E	0.27	0.02	46	1	1	0.29	0.02	37	2	1
389	Y	0.28	0.01	49	1	1	0.30	0.02	42	2	0
390	E	0.28	0.01	47	1	1	0.31	0.02	43	2	1
391	M	0.28	0.01	49	1	1	0.33	0.04	43	4	1
392	R	0.33	0.01	40.5	0.4	1	0.33	0.01	43	1	1
393	N	0.26	0.01	47	1	1	0.31	0.01	42	1	1
400	L	0.26	0.05	49	4	0	0.20	0.07	43	8	0
401	L	0.28	0.02	47	3	1	0.37	0.06	53	5	0
402	L	0.26	0.01	46	1	1	0.29	0.02	41	1	0
403	D	0.53	0.01	34	1	0	0.53	0.01	30.4	0.3	0
404	K	0.34	0.01	38	1	1	0.35	0.02	36	1	1
405	G	0.32	0.01	43	1	1	0.35	0.02	38	1	1

406	V	0.39	0.01	43	1	0	0.41	0.01	43	2	0
407	Y	0.24	0.01	53	1	1	0.23	0.02	46	1	1
408	G	0.17	0.07	45	5	0	0.24	0.07	49	7	0
415	H	0.28	0.01	48	2	1	0.24	0.03	45	3	1
416	G	0.25	0.02	45	1	1	0.28	0.01	39	2	1
417	Y	0.35	0.01	49	1	1	0.34	0.02	48	3	1
418	E	0.38	0.03	43	1	0	0.33	0.03	64	5	0
419	N	0.30	0.02	42	1	0	0.32	0.02	41	2	0
421	G	0.38	0.01	41	1	0	0.42	0.02	40	2	0
422	N	0.33	0.01	45	1	0	0.36	0.01	38	2	0
423	S	0.30	0.03	45	3	0	0.37	0.06	41	7	0
424	F	0.30	0.03	60	1	0	0.25	0.01	50	3	1
425	M	0.23	0.02	44	1	1	0.27	0.02	43	3	1
426	V	0.26	0.01	44	2	0	0.46	0.06	52	8	0
428	V	0.25	0.07	56	4	0	0.25	0.12	45	11	0
429	D	0.29	0.01	53	2	1	0.33	0.02	46	2	1
430	A	0.30	0.01	47	1	0	0.34	0.02	37	3	0
432	N	0.26	0.01	45	1	0	0.29	0.02	40	1	0
434	Y	0.30	0.01	42	1	0	0.35	0.01	34	2	0
435	R	0.39	0.03	43	3	0	0.46	0.04	35	5	0
445	I	0.27	0.02	53	2	1	0.31	0.02	48	1	1
446	L	0.32	0.01	45	1	1	0.36	0.03	41	1	1
448	L	0.32	0.02	46	2	1	0.32	0.03	43	2	1
449	M	0.33	0.01	44	1	0	0.36	0.03	40	1	0
450	Q	0.30	0.02	46	2	1	0.33	0.03	38.3	0.4	1
451	E	0.33	0.02	46	2	0	0.32	0.05	40	1	0
452	K	0.36	0.01	42	1	0	0.34	0.01	36	1	0
453	E	0.33	0.02	45	1	1	0.43	0.03	41	2	1
454	T	0.67	0.02	34	1	0	0.64	0.04	25	2	0
455	G	0.27	0.03	50	2	0	0.31	0.03	42	3	1
456	L	0.39	0.02	10	4	0	0.34	0.05	52	10	0
457	N	0.24	0.01	53	1	1	0.27	0.02	47	2	1
458	V	0.25	0.01	46	1	0	0.25	0.02	41	1	1
459	F	0.23	0.01	55	2	0	0.23	0.02	45	1	1
460	L	0.24	0.01	48	1	0	0.23	0.01	43	1	1
461	L	0.27	0.01	48	1	1	0.27	0.02	45	1	1
462	D	0.26	0.01	49	1	1	0.30	0.02	45	2	1
463	M	0.26	0.01	46	1	1	0.34	0.01	40	1	1
464	C	0.33	0.02	42	1	0	0.30	0.01	47	1	0
465	R	0.32	0.03	55	1	0	0.35	0.04	43	7	0
471	D	0.37	0.01	38.9	0.3	0	0.48	0.02	39.3	0.3	0
472	D	0.64	0.01	32	1	0	0.67	0.01	33	3	0
473	T	0.55	0.02	39	1	0	0.66	0.02	24	6	0
474	I	0.67	0.06	38	2	0	0.75	0.12	16	40	0
476	I	0.82	0.04	19	1	0	0.97	0.06	19	3	0
487	V	0.24	0.01	50	1	1	0.26	0.01	42	2	1
488	F	0.24	0.02	50	2	1	0.22	0.03	50	1	0
489	G	0.24	0.01	50	1	1	0.24	0.02	43	1	0
490	Y	0.25	0.03	55	3	0	0.22	0.04	42	3	0

491	A	0.23	0.04	48	3	0	0.32	0.05	54	4	0
496	A	0.55	0.02	33	1	0	0.64	0.03	35	7	0
497	E	0.59	0.02	34	1	0	0.64	0.02	28	4	0
498	A	0.71	0.02	28	1	0	0.76	0.01	26	4	0
499	F	0.61	0.02	34.1	0.3	0	0.69	0.02	64	4	0
500	E	0.73	0.01	22.2	0.2	0	0.79	0.01	29	1	0
501	I	0.73	0.02	27.6	0.4	0	0.78	0.01	26	1	0
502	Q	0.76	0.02	28.4	0.3	0	0.84	0.02	25	6	0
506	L	0.67	0.02	24.6	0.2	0	0.68	0.01	28	3	0
507	A	0.59	0.01	26.7	0.3	0	0.62	0.01	24	1	0
508	N	0.35	0.01	39.3	0.4	0	0.43	0.01	33	1	0
509	G	0.28	0.02	47	2	0	0.33	0.02	41	4	0
510	I	0.34	0.02	47	2	0	0.39	0.05	41	5	0
512	M	0.29	0.05	50	2	0	0.32	0.04	-	-	0
513	K	0.28	0.02	46	1	1	0.35	0.02	43	4	0
514	F	0.29	0.02	48	2	0	0.33	0.03	39	2	0
515	L	0.34	0.01	45	1	1	0.35	0.03	44	2	1
516	K	0.27	0.02	48	2	0	0.28	0.03	41	1	0
517	D	0.36	0.04	39	2	0	0.41	0.05	38	2	0
518	R	0.34	0.03	41	3	0	0.36	0.04	38	1	0
519	L	0.31	0.01	45	2	0	0.27	0.03	40	2	0
520	L	0.30	0.02	43	1	0	0.29	0.03	39	2	0
521	E	0.33	0.01	45	1	0	0.34	0.02	41	1	0
522	D	0.33	0.01	40	1	0	0.35	0.01	33	1	0
523	K	0.32	0.01	50	1	0	0.37	0.01	40	4	0
524	K	0.32	0.01	49	1	1	0.34	0.01	43	2	1
525	I	0.34	0.02	44	1	1	0.37	0.01	41	1	1
526	T	0.26	0.03	48	4	0	0.39	0.05	39	10	0
527	V	0.33	0.02	43	1	1	0.27	0.02	34	1	0
528	L	0.18	0.19	11	15	0	0.64	0.30	32	20	0
529	L	0.32	0.01	44	1	1	0.36	0.01	42	1	1
530	D	0.34	0.02	43	1	1	0.37	0.02	40	2	1
531	E	0.42	0.01	33	1	0	0.45	0.02	39	1	1
532	V	0.32	0.01	44	1	1	0.31	0.02	40	1	1
533	A	0.36	0.01	41	1	0	0.36	0.02	40	1	0
534	E	0.35	0.01	42	1	0	0.39	0.01	39	1	0
536	M	0.35	0.02	45	1	0	0.36	0.02	42	1	0
537	G	0.36	0.02	45	1	0	0.39	0.01	36	3	0
538	K	0.38	0.01	44	1	0	0.42	0.02	37	3	0
545	K	0.58	0.02	35	1	0	0.64	0.01	50	8	0
546	Q	0.23	0.02	52	2	0	0.23	0.02	51	3	0
549	E	0.30	0.02	49	1	1	0.27	0.02	44	1	1
550	I	0.28	0.01	54	1	0	0.32	0.02	48	2	1
551	R	0.27	0.01	50	1	1	0.25	0.02	45	2	1
552	S	0.27	0.01	49	1	0	0.32	0.01	34	6	0
553	S	0.35	0.02	54	2	0	0.45	0.04	45	9	0
558	R	0.42	0.01	35	2	1	0.48	0.04	29	2	0
559	A	0.29	0.01	46	1	0	0.31	0.02	43	2	1
560	L	0.24	0.03	46	3	0	0.32	0.06	12	20	0

561	T	0.30	0.02	55	2	0	0.28	0.03	44	5	1
564	I	0.27	0.01	45.7	0.3	0	0.26	0.02	39.6	0.4	0
565	Q	0.35	0.01	42.7	0.4	0	0.37	0.02	36.6	0.4	0
566	G	0.62	0.01	27.6	0.2	0	0.67	0.01	20	4	0
567	T	0.72	0.01	22.1	0.3	0	0.74	0.02	15	9	0
572	E	0.68	0.01	26.0	0.3	0	0.73	0.02	25	2	0
573	S	0.67	0.01	27.0	0.3	0	0.71	0.01	9	5	0
574	L	0.7	0.01	28.1	0.2	0	0.73	0.01	23	1	0
575	V	0.5	0.01	39	1	0	0.52	0.01	34	1	1
576	R	0.51	0.02	36	1	0	0.53	0.03	37	4	1
577	N	0.43	0.02	43	1	1	0.47	0.01	29	3	0
578	L	0.41	0.01	40	1	1	0.49	0.01	35	3	1
579	Q	0.38	0.01	48	0.4	0	0.43	0.02	48	3	1
580	W	0.31	0.01	43	0.2	1	0.34	0.01	40	1	0
581	A	0.30	0.02	49	1	1	0.38	0.02	45	3	1
582	K	0.34	0.02	49	1	1	0.35	0.05	53	6	0
583	A	0.31	0.02	54	1	1	0.34	0.02	51	2	0
585	E	0.32	0.01	40.7	0.4	0	0.35	0.01	36	1	1
586	L	0.37	0.01	38.0	0.1	1	0.36	0.02	33	1	1
588	E	0.35	0.01	38.5	0.3	1	0.31	0.01	36.5	0.3	1
589	S	0.29	0.01	41.7	0.3	1	0.28	0.02	34	1	1
590	M	0.30	0.01	45.4	0.4	1	0.31	0.02	38	1	1
591	C	0.29	0.01	41.1	0.3	1	0.32	0.01	37	1	1
592	L	0.29	0.01	42	2	1	0.35	0.05	33	2	0
593	K	0.29	0.01	40.9	0.2	1	0.29	0.01	37	1	1
594	F	0.29	0.01	41.7	0.3	1	0.3	0.01	35.7	0.4	1
595	D	0.28	0.01	46	1	1	0.29	0.01	36	1	1
597	G	0.28	0.01	43	1	1	0.28	0.02	41	2	1
598	V	0.26	0.03	45	2	0	0.33	0.05	35	9	0
599	Q	0.26	0.01	44	1	1	0.28	0.02	40	1	1
600	I	0.33	0.02	44	2	0	0.26	0.03	39	3	1
601	Q	0.29	0.06	42	5	0	0.26	0.10	44	10	0
602	L	0.31	0.01	43	1	1	0.31	0.02	37	2	0
603	G	0.17	0.06	57	6	0	0.40	0.06	34	8	0
604	F	0.26	0.01	43.5	0.3	1	0.27	0.02	38	1	1
605	A	0.24	0.03	40	2	0	0.24	0.06	33	2	0
606	A	0.29	0.01	43.2	0.4	0	0.29	0.02	36.2	0.2	0
616	T	0.27	0.01	44	1	1	0.29	0.02	36	2	1
617	S	0.28	0.02	44	1	1	0.24	0.03	41	2	1
618	I	0.32	0.01	42	1	0	0.32	0.02	37	2	0
619	V	0.34	0.01	38	1	1	0.34	0.02	36	1	1
620	Y	0.33	0.01	43	1	1	0.34	0.02	39	1	1
621	K	0.32	0.01	41.5	0.3	0	0.33	0.02	36.4	0.3	1
624	E	0.29	0.01	43	1	1	0.33	0.02	37	1	1
625	I	0.30	0.01	46	1	1	0.31	0.02	41	1	1
626	I	0.33	0.01	39.3	0.4	1	0.38	0.02	42	1	1
627	M	0.40	0.02	43	1	1	0.52	0.03	39	1	1
628	C	0.36	0.01	42.9	0.4	1	0.36	0.01	39	2	1
629	D	0.29	0.01	48	1	1	0.31	0.01	45	2	0

630	A	0.28	0.01	41	1	1	0.25	0.02	34	1	0
631	Y	0.38	0.02	42	1	0	0.50	0.06	37	4	0
632	V	0.28	0.01	44	2	1	0.31	0.03	39	1	1
633	T	0.27	0.01	46.4	0.4	0	0.29	0.01	39	1	1
634	D	0.30	0.01	41.7	0.3	1	0.33	0.01	38	1	1
635	F	0.28	0.01	44	1	1	0.3	0.01	37	1	0
637	L	0.31	0.01	43	1	0	0.34	0.01	38	2	0
638	D	0.32	0.01	49	1	0	0.35	0.01	37	2	0
639	L	0.26	0.01	47	1	0	0.27	0.01	42	1	0
640	D	0.36	0.01	41.0	0.3	0	0.38	0.02	36	1	0
641	I	0.35	0.01	43.0	0.3	0	0.35	0.02	37	1	0
642	D	0.38	0.01	42	1	0	0.37	0.02	33	1	0
644	K	0.34	0.01	45	0.3	0	0.33	0.02	39	1	0
645	D	0.33	0.02	39.8	1	0	0.32	0.03	37	1	0
646	A	0.36	0.01	42	1	0	0.38	0.02	35	1	1
647	N	0.24	0.01	48	1	1	0.28	0.01	37	2	0
648	K	0.26	0.01	51	1	0	0.28	0.01	42	2	1
649	G	0.29	0.01	40	1	1	0.31	0.02	36	2	1
650	T	0.29	0.01	46	1	0	0.32	0.01	37	5	0
652	E	0.30	0.01	47	1	1	0.37	0.02	38	30	0
653	E	0.26	0.01	48	1	0	0.31	0.01	40	4	1
654	T	0.30	0.03	42	2	0	0.30	0.04	22	50	0
655	G	0.30	0.02	50	1	0	0.31	0.03	43	10	0
656	S	0.26	0.03	55	3	0	0.30	0.03	-	-	0
657	Y	0.25	0.04	50	3	0	0.38	0.03	33	10	0
658	L	0.37	0.02	43	2	0	0.40	0.03	35	7	0
659	V	0.28	0.02	47	1	0	0.29	0.03	33	4	0
661	K	0.37	0.01	50	1	0	0.39	0.02	49	8	0
662	D	0.43	0.01	41	1	0	0.46	0.02	35	2	0
663	L	0.34	0.01	41.0	0.3	0	0.38	0.02	35	2	0
665	K	0.33	0.01	41.2	0.3	0	0.34	0.01	37	1	1
666	H	0.32	0.01	45	1	1	0.33	0.01	29	3	0
667	C	0.27	0.01	42	2	1	0.31	0.00	49	2	1
668	L	0.32	0.01	30	1	0	0.3	0.02	41	1	1
669	Y	0.26	0.01	45	1	1	0.26	0.01	40	2	1
670	T	0.24	0.01	43	1	1	0.25	0.02	34	2	1
671	R	0.26	0.01	46	1	1	0.28	0.01	39	1	1
672	L	0.28	0.01	40.8	0.4	1	0.29	0.02	34.4	0.3	0
673	S	0.30	0.04	41	2	0	0.37	0.05	30	3	0
674	S	0.24	0.03	59	4	0	0.22	0.05	52	3	0
675	L	0.33	0.02	41	1	1	0.38	0.02	39	1	1
676	Q	0.29	0.04	48	1	0	0.30	0.06	39	4	0
677	K	0.4	0.01	41	1	1	0.41	0.02	39	2	0
678	L	0.29	0.01	47	1	1	0.29	0.02	41	1	1
679	K	0.37	0.01	42	1	0	0.38	0.01	36	2	1
680	E	0.55	0.01	33.1	0.2	0	0.59	0.01	28	1	0
681	H	0.46	0.02	34.7	0.4	1	0.49	0.01	30	1	1
682	L	0.29	0.01	42	1	1	0.28	0.02	37.3	0.3	1
683	V	0.28	0.01	43	1	0	0.26	0.01	37	1	1

688	L	0.25	0.04	48	5	0	0.13	0.04	23	20	0
689	S	0.30	0.01	47	1	1	0.31	0.01	44	1	1
690	Y	0.32	0.01	49	1	1	0.34	0.02	40	1	1
691	Q	0.26	0.01	46	1	1	0.30	0.01	40	1	1
692	Y	0.31	0.01	43.5	0.4	1	0.32	0.02	39	1	1
693	S	0.34	0.01	39.8	0.4	1	0.34	0.01	32	1	0
694	G	0.36	0.01	38.0	0.4	1	0.39	0.01	28	5	0
695	L	0.37	0.01	43.5	0.3	1	0.40	0.01	39	1	1
696	E	0.43	0.02	37	1	1	0.44	0.01	31	1	1
697	D	0.47	0.01	32.4	0.2	0	0.48	0.02	26	1	0
698	T	0.38	0.01	34.2	0.3	0	0.38	0.02	28.8	0.4	0
699	V	0.34	0.01	40	1	0	0.35	0.02	32	1	1
700	E	0.34	0.01	41.9	0.3	0	0.29	0.01	37	1	0
701	D	0.31	0.01	42.8	0.3	0	0.33	0.01	34	1	0
702	K	0.31	0.01	43	1	1	0.30	0.02	36	1	1
703	Q	0.26	0.01	44.2	0.3	1	0.24	0.02	40	1	1
704	E	0.3	0.01	44.3	0.2	0	0.31	0.01	38	1	0
705	V	0.32	0.01	42	1	0	0.27	0.03	38	2	0
706	N	0.29	0.01	40.3	0.2	0	0.27	0.01	39.4	0.3	0
707	V	0.30	0.01	42	1	0	0.34	0.01	38	1	0
708	G	0.27	0.02	53	2	1	0.24	0.02	43	1	1
709	K	0.30	0.01	40.8	0.4	0	0.36	0.01	37	1	0
711	L	0.30	0.01	47	1	0	0.32	0.01	39	1	1
712	I	0.55	0.05	27	1	0	0.73	0.06	35	3	0
713	A	0.34	0.01	48	1	0	0.37	0.02	48	3	0
714	K	0.33	0.02	45	1	1	0.32	0.02	39	2	0
715	L	0.31	0.01	51	1	1	0.31	0.03	42	2	1
716	D	0.37	0.01	48	1	0	0.41	0.01	41	2	0
717	M	0.34	0.01	47	1	0	0.40	0.01	34	3	0
718	H	0.41	0.03	47	2	0	0.44	0.03	37	7	0
719	R	0.46	0.02	42	1	0	0.52	0.02	40	4	0

Table S2. NOE ^{1}H-^{15}N relaxations for 800 MHz

Residue	Type	800 MHz	
		NOE	error
340	A	0.71	0.02
341	K	0.80	0.08
342	D	0.96	0.09
343	K	0.83	0.04
352	N	0.81	0.09
353	Y	0.72	0.02
354	R	0.76	0.04
355	E	1.03	0.16
356	H	0.74	0.05
358	K	0.73	0.02
359	L	0.69	0.03
361	A	0.71	0.13
363	L	0.78	0.06

364	V	0.66	0.10
365	D	0.85	0.09
366	V	0.72	0.03
367	Y	0.78	0.07
368	E	0.82	0.06
369	L	0.91	0.06
374	R	0.83	0.04
375	Q	0.94	0.07
376	L	0.78	0.07
377	D	0.82	0.05
378	F	0.56	0.10
379	K	0.78	0.04
380	V	0.65	0.05
381	V	0.77	0.09
386	L	0.82	0.06
387	T	0.82	0.05
388	E	0.77	0.06
389	Y	0.94	0.06
390	E	0.74	0.07
391	M	0.83	0.10
392	R	0.81	0.05
396	D	0.91	0.18
400	L	0.53	0.24
401	L	1.06	0.26
402	L	0.84	0.05
403	D	0.32	0.03
404	K	0.59	0.04
405	G	0.66	0.04
406	V	0.78	0.04
407	Y	0.75	0.06
414	G	0.57	0.12
415	H	0.87	0.10
416	G	0.85	0.07
417	Y	1.16	0.15
418	E	0.67	0.09
419	N	0.79	0.05
421	G	0.59	0.05
422	N	0.81	0.05
423	S	0.73	0.17
424	F	0.71	0.08
425	M	0.95	0.12
426	V	0.81	0.06
429	D	0.81	0.07
430	A	0.83	0.05
432	N	0.66	0.04
434	Y	0.68	0.03
435	R	0.54	0.12
444	N	0.80	0.03
445	I	1.07	0.09

446	L	0.90	0.08
447	K	0.64	0.05
448	L	0.87	0.09
449	M	0.72	0.07
450	Q	0.79	0.08
451	E	0.67	0.08
452	K	0.70	0.04
453	E	0.63	0.05
454	T	0.52	0.05
455	G	0.67	0.07
456	L	0.65	0.04
457	N	0.75	0.06
458	V	0.75	0.06
459	F	0.99	0.07
460	L	0.70	0.05
461	L	0.81	0.06
462	D	0.82	0.07
463	M	0.79	0.03
472	D	0.43	0.03
473	T	0.48	0.06
476	I	0.22	0.08
487	V	1.03	0.09
488	F	0.95	0.10
489	G	0.94	0.06
490	Y	0.85	0.15
491	A	0.82	0.12
495	G	0.48	0.08
496	A	0.53	0.05
497	E	0.49	0.05
498	A	0.47	0.03
499	F	0.50	0.03
500	E	0.43	0.02
501	I	0.44	0.03
502	Q	0.47	0.03
507	A	0.30	0.02
508	N	0.60	0.03
509	G	0.98	0.09
510	I	0.81	0.10
512	M	0.57	0.12
513	K	0.85	0.06
514	F	0.85	0.08
515	L	0.89	0.11
516	K	0.74	0.06
518	R	0.85	0.11
519	L	0.70	0.09
520	L	0.78	0.06
521	E	0.61	0.05
522	D	0.78	0.03
523	K	0.84	0.07

524	K	0.76	0.06
525	I	0.81	0.06
526	T	0.93	0.18
527	V	0.64	0.09
528	L	0.67	0.09
529	L	0.79	0.07
530	D	0.75	0.05
531	E	0.64	0.02
532	V	0.76	0.07
533	A	0.86	0.08
534	E	0.73	0.05
535	D	0.61	0.07
536	M	0.72	0.06
537	G	0.70	0.07
538	K	0.83	0.09
539	C	0.63	0.06
540	H	0.93	0.08
541	L	0.73	0.02
545	K	0.53	0.04
546	Q	0.93	0.11
549	E	0.71	0.07
550	I	0.65	0.06
551	R	0.86	0.06
552	S	0.77	0.05
558	R	0.29	0.09
559	A	0.94	0.06
560	L	0.84	0.18
561	T	0.83	0.10
562	D	0.77	0.05
564	I	0.67	0.03
565	Q	0.74	0.03
566	G	0.48	0.02
567	T	0.42	0.02
572	E	0.41	0.02
573	S	0.44	0.03
574	L	0.50	0.02
575	V	0.54	0.03
576	R	0.54	0.06
578	L	0.77	0.03
579	Q	0.69	0.05
580	W	0.74	0.03
581	A	0.71	0.06
582	K	0.81	0.12
583	A	0.90	0.08
585	E	0.72	0.02
586	L	0.70	0.03
588	E	0.79	0.05
589	S	0.56	0.02
590	M	0.73	0.04

591	C	0.61	0.03
592	L	0.79	0.02
593	K	0.64	0.03
594	F	0.69	0.03
595	D	0.83	0.05
597	G	0.87	0.04
598	V	0.77	0.20
599	Q	0.72	0.06
600	I	0.66	0.15
601	Q	0.34	0.25
602	L	0.71	0.04
603	G	0.55	0.21
604	F	0.78	0.03
605	A	0.52	0.16
606	A	0.81	0.03
616	T	0.79	0.05
617	S	0.75	0.07
618	I	0.65	0.03
619	V	0.73	0.05
620	Y	0.82	0.06
621	K	0.73	0.02
624	E	0.74	0.04
625	I	0.74	0.04
626	I	0.66	0.05
627	M	0.41	0.04
628	C	0.68	0.03
629	D	0.66	0.05
630	A	0.66	0.03
631	Y	0.76	0.07
632	V	0.76	0.07
633	T	0.80	0.03
634	D	0.76	0.03
635	F	0.65	0.03
637	L	0.76	0.04
638	D	0.63	0.05
639	L	0.74	0.05
640	D	0.57	0.03
641	I	0.61	0.02
642	D	0.45	0.02
644	K	0.66	0.03
645	D	0.66	0.04
646	A	0.60	0.03
647	N	0.81	0.06
648	K	0.84	0.05
649	G	0.76	0.04
650	T	0.92	0.06
652	E	0.81	0.07
653	E	0.69	0.07
654	T	0.66	0.10

655	G	0.72	0.08
656	S	0.50	0.10
657	Y	0.81	0.20
658	L	0.74	0.11
659	V	0.66	0.05
661	K	0.56	0.05
662	D	0.51	0.04
663	L	0.53	0.04
665	K	0.62	0.03
666	H	0.57	0.05
667	C	0.66	0.04
668	L	0.67	0.04
669	Y	0.84	0.05
670	T	0.74	0.04
671	R	0.83	0.04
672	L	0.79	0.02
673	S	0.59	0.11
674	S	0.66	0.11
675	L	0.83	0.05
676	Q	1.01	0.12
677	K	0.73	0.05
678	L	0.82	0.05
679	K	0.66	0.05
680	E	0.42	0.03
681	H	0.26	0.02
682	L	0.72	0.04
683	V	0.73	0.04
685	T	0.81	0.09
689	S	0.84	0.04
690	Y	0.75	0.04
691	Q	0.80	0.03
692	Y	0.51	0.03
693	S	0.56	0.03
694	G	0.53	0.03
695	L	0.48	0.03
696	E	0.18	0.02
697	D	0.00	0.02
698	T	0.28	0.02
699	V	0.61	0.03
700	E	0.61	0.02
701	D	0.69	0.02
702	K	0.64	0.03
703	Q	0.75	0.03
704	E	0.70	0.01
705	V	0.80	0.04
706	N	0.75	0.03
707	V	0.77	0.03
708	G	0.81	0.08
709	K	0.68	0.02

711	L	0.73	0.03
713	A	0.88	0.07
714	K	0.88	0.07
715	L	1.01	0.10
716	D	0.68	0.04
717	M	0.88	0.07
718	H	0.65	0.08
719	R	0.63	0.04

Table S3. **Ratio of intensities (I^{β}/I^{α}) used in Eq. (5) measured in TROSY/anti-TROSY experiments at 900MHz for MALT1(PCASP-Ig3)$_{339\text{–}719}$**

Residue	Type	900 MHz	
		I^{β}/I^{α}	Error (σ)
340	A	0.43	0.02
341	K	0.42	0.02
342	D	0.45	0.05
343	K	0.36	0.17
352	N	0.46	0.03
353	Y	0.48	0.02
354	R	0.55	0.03
355	E	0.62	0.07
356	H	0.44	0.04
358	K	0.48	0.02
359	L	0.48	0.05
361	A	0.53	0.20
363	L	0.41	0.06
364	V	0.62	0.03
365	D	0.54	0.05
366	V	0.43	0.01
367	Y	0.42	0.01
368	E	0.48	0.02
369	L	0.43	0.04
374	R	0.47	0.02
375	Q	0.42	0.01
376	L	0.48	0.02
377	D	0.43	0.02
378	F	0.45	0.02
379	K	0.41	0.03
380	V	0.45	0.02
381	V	0.42	0.02
386	L	0.69	0.03
387	T	0.45	0.02
388	E	0.49	0.04
389	Y	0.43	0.01
390	E	0.40	0.02
391	M	0.41	0.05
392	R	0.56	0.02

400	L	0.47	0.05
401	L	0.43	0.10
402	L	0.45	0.02
403	D	0.55	0.04
404	K	0.57	0.04
405	G	0.50	0.02
406	V	0.69	0.03
407	Y	0.41	0.01
408	G	0.45	0.02
414	G	0.46	0.11
415	H	0.55	0.04
416	G	0.55	0.05
417	Y	0.51	0.13
418	E	0.65	0.03
419	N	0.49	0.02
421	G	0.57	0.09
422	N	0.46	0.02
423	S	0.61	0.27
424	F	0.41	0.02
425	M	0.42	0.07
426	V	0.48	0.03
428	V	1.16	0.43
429	D	0.40	0.02
430	A	0.46	0.02
432	N	0.41	0.01
434	Y	0.47	0.03
435	R	0.66	0.13
444	N	0.44	0.01
445	I	0.42	0.09
446	L	0.44	0.02
448	L	0.46	0.02
449	M	0.42	0.03
450	Q	0.47	0.02
451	E	0.42	0.01
452	K	0.49	0.02
453	E	0.48	0.05
455	G	0.45	0.05
456	L	0.52	0.03
457	N	0.41	0.02
458	V	0.45	0.02
459	F	0.38	0.03
460	L	0.48	0.02
461	L	0.46	0.02
462	D	0.33	0.04
463	M	0.46	0.02
472	D	0.72	0.04
473	T	0.68	0.09
474	I	0.47	0.05
476	I	0.81	0.04

487	V	0.37	0.01
488	F	0.38	0.05
489	G	0.49	0.02
490	Y	0.57	0.12
491	A	0.38	0.28
496	A	0.74	0.07
497	E	0.47	0.05
498	A	0.78	0.04
499	F	0.62	0.02
500	E	0.74	0.04
501	I	0.67	0.03
502	Q	0.73	0.04
507	A	0.64	0.03
508	N	0.49	0.02
509	G	0.59	0.02
510	I	0.51	0.06
512	M	0.98	0.36
513	K	0.34	0.05
514	F	0.49	0.06
515	L	0.43	0.21
516	K	0.55	0.06
517	D	0.66	0.12
518	R	0.67	0.03
519	L	0.40	0.04
520	L	0.53	0.05
521	E	0.44	0.04
522	D	0.46	0.03
523	K	0.49	0.04
524	K	0.46	0.02
525	I	0.44	0.07
526	T	0.44	0.01
527	V	0.46	0.11
528	L	0.44	0.02
529	L	0.60	0.03
530	D	0.47	0.02
531	E	0.52	0.03
532	V	0.47	0.06
533	A	0.63	0.05
534	E	0.56	0.03
535	D	0.57	0.02
536	M	0.41	0.04
537	G	0.55	0.04
539	C	0.43	0.02
540	H	0.47	0.02
541	L	0.47	0.02
545	K	0.61	0.04
546	Q	0.46	0.04
549	E	0.41	0.02
550	I	0.45	0.09

551	R	0.44	0.05
552	S	0.46	0.05
558	R	0.53	0.03
559	A	0.45	0.04
560	L	0.57	0.04
561	T	0.44	0.04
562	D	0.42	0.02
564	I	0.43	0.03
565	Q	0.44	0.02
566	G	0.69	0.05
567	T	0.77	0.07
572	E	0.68	0.03
573	S	0.71	0.03
574	L	0.62	0.03
575	V	0.52	0.03
576	R	0.65	0.03
578	L	0.51	0.02
579	Q	0.46	0.02
580	W	0.47	0.02
581	A	0.45	0.03
582	K	0.44	0.02
583	A	0.40	0.02
585	E	0.47	0.02
586	L	0.49	0.02
588	E	0.50	0.03
589	S	0.46	0.02
590	M	0.44	0.02
591	C	0.50	0.03
592	L	0.47	0.02
593	K	0.44	0.02
594	F	0.46	0.02
595	D	0.49	0.02
597	G	0.50	0.02
598	V	0.40	0.02
599	Q	0.44	0.01
600	I	0.46	0.02
601	Q	0.43	0.07
602	L	0.43	0.03
603	G	0.44	0.02
604	F	0.47	0.02
605	A	0.42	0.04
606	A	0.45	0.03
616	T	0.49	0.02
617	S	0.45	0.02
618	I	0.44	0.01
619	V	0.51	0.03
620	Y	0.45	0.02
621	K	0.42	0.02
624	E	0.50	0.03

625	I	0.42	0.02
626	I	0.47	0.02
627	M	0.46	0.02
628	C	0.50	0.03
629	D	0.44	0.01
630	A	0.45	0.02
631	Y	0.41	0.01
632	V	0.50	0.03
633	T	0.44	0.02
635	F	0.49	0.02
637	L	0.48	0.02
638	D	0.47	0.02
639	L	0.42	0.03
640	D	0.46	0.02
641	I	0.48	0.02
642	D	0.45	0.02
644	K	0.44	0.02
645	D	0.51	0.02
646	A	0.45	0.03
647	N	0.47	0.02
648	K	0.43	0.02
649	G	0.53	0.03
650	T	0.52	0.03
652	E	0.49	0.02
653	E	0.48	0.02
654	T	0.52	0.05
655	G	0.47	0.02
656	S	0.42	0.10
657	Y	0.52	0.05
658	L	0.52	0.07
659	V	0.56	0.18
660	S	0.70	1.22
662	D	0.48	0.03
663	L	0.47	0.03
665	K	0.49	0.02
666	H	0.51	0.03
667	C	0.49	0.02
668	L	0.56	0.02
669	Y	0.47	0.03
670	T	0.49	0.02
671	R	0.43	0.01
672	L	0.47	0.02
673	S	0.48	0.06
674	S	0.42	0.02
675	L	0.53	0.02
676	Q	0.50	0.03
677	K	0.48	0.02
678	L	0.41	0.04
679	K	0.50	0.02

680	E	0.56	0.03
681	H	0.58	0.03
682	L	0.46	0.02
683	V	0.44	0.04
688	L	0.45	0.05
689	S	0.42	0.02
690	Y	0.43	0.01
691	Q	0.43	0.01
692	Y	0.48	0.02
693	S	0.49	0.02
694	G	0.64	0.09
695	L	0.44	0.02
696	E	0.58	0.02
697	D	0.55	0.02
698	T	0.55	0.03
699	V	0.48	0.02
700	E	0.43	0.02
701	D	0.43	0.03
702	K	0.43	0.03
703	Q	0.41	0.02
704	E	0.42	0.01
705	V	0.46	0.02
706	N	0.44	0.02
707	V	0.46	0.02
708	G	0.43	0.01
709	K	0.52	0.06
711	L	0.44	0.02
713	A	0.42	0.01
714	K	0.44	0.05
715	L	0.44	0.02
716	D	0.47	0.02
717	M	0.48	0.04
718	H	0.52	0.05
719	R	0.53	0.04

Table S4 The free energy change (ΔΔG) induced by a single W580A mutation in the X-ray and AF structures identifying destabilizing effect of the mutation relative to the ground state (positive ΔΔ*G*).

**X-ray and AF structures with W580 faces inward into the allosteric site.**	**ΔΔG (kcal/mol)**	**X-ray and AF structures with W580 faces outward the allosteric site.**	**ΔΔG (kcal/mol)**
3v55 A	1.78	7A41	1.23
3uoa C	1.99	3v4L	1.39
3UO8 C	1.99	6yn9	1.34
4I1P A	1.84	6H4A	1.27
6YN8 A	1.65	7AK0	1.07
AF model_1	1.84	7AK1	1.34
		6F71	1.14

		4i1r	0.9
		AF model 3	1.4
Mean / standard deviation	1.85/0.13		1.18/0.17

Table S5. List of acquisition parameters used for the NMR experiments

Experiments	Maximum evolution time, (ms)/ carrier frequency (ppm)/sweep width (ppm)			D1 (s)	Scans	NUS %	Time (h)
	F3	F2	F1				
3D ^{1}H–^{15}N SF-TROSY-NOESY[(a)]	79.9(^{1}H)/4.67/ 16.0	27.4(^{15}N)/11 8.0/36.0	28.4(^{1}H)/4. 67/11.0	0.5	16	23	68
4D ^{13}C-SF-HMQC NOESY[(b)]	F4 81.0(^{1}H)/4.7/ 14.0	F3/F2 9.8(^{13}C)/ 17.0/18.0	F1 19.7(^{1}H)/4. 7/1.8	0.7	8	10.5	84
3D pseudo TROSY-T1 R1[(a)] 3D trt1etf3gpsitc3d.3 R1[(b)]	119.8(^{1}H)/4.7/ 16.0<br>70.9(^{1}H)/4.7/ 16.0	37.7(^{15}N)/11 8.0/ 36.0<br>37.6(^{15}N)/11 8.0/ 32.0	-	1.2<br>1.2	40<br>40	44<br>50	28<br>33
3D pseudo TROSY-T2 R2[(a)] 3D trt2etf3gpsitc3d.3 R2[(b)] 3D	79.8(^{1}H)/4.7/ 16.0<br>70.9(^{1}H)/4.7/ 16.0	37.7(^{15}N)/11 8.0/ 36.0<br>37.6(^{15}N)/11 8.0/ 32.0	-	1.5<br>1.5	56<br>56	45<br>54	23<br>33
NOE[(a)] 3D trnoeetf3gpsi3d.3 3D pseudo TROSY-NOE	79.8(^{1}H)/4.7/ 16.0	34.2(^{15}N)/11 8.0/ 36.0	-	6	40	-	27
CCR-^{15}N[(b)]	79.8(^{1}H)/4.7/ 16.0	37.6(^{15}N)/11 8.0/ 32.0	-	1.5	64	-	26

a) [(a)] Experiments performed on an 800MHz spectrometer.

b) [(b)] Experiments on 900MHz spectrometer
